# Characteristics of the Protein Complexes and Pores Formed by *Bacillus cereus* Hemolysin BL

**DOI:** 10.3390/toxins12110672

**Published:** 2020-10-24

**Authors:** Nadja Jessberger, Richard Dietrich, Kristina Schauer, Stefanie Schwemmer, Erwin Märtlbauer, Roland Benz

**Affiliations:** 1 Department of Veterinary Sciences, Faculty of Veterinary Medicine, Ludwig-Maximilians-Universität München, Schönleutnerstr. 8, 85764 Oberschleißheim, Germany; r.dietrich@mh.vetmed.uni-muenchen.de (R.D.); kristina.schauer@mh.vetmed.uni-muenchen.de (K.S.); schwemmer.stefanie@web.de (S.S.); e.maertlbauer@mh.vetmed.uni-muenchen.de (E.M.); 2 Life Sciences & Chemistry, Jacobs-University Bremen, Campus Ring 1, 28759 Bremen, Germany; r.benz@jacobs-university.de

**Keywords:** *Bacillus cereus*, hemolysin BL, planar lipid bilayer, protein complexes, heat stability, pore formation, cytotoxicity

## Abstract

*Bacillus cereus* Hemolysin BL is a tripartite toxin responsible for a diarrheal type of food poisoning. Open questions remain regarding its mode of action, including the extent to which complex formation prior to cell binding contributes to pore-forming activity, how these complexes are composed, and the properties of the pores formed in the target cell membrane. Distinct complexes of up to 600 kDa were found on native gels, whose structure and size were primarily defined by Hbl B. Hbl L1 and L2 were also identified in these complexes using Western blotting and an LC-MS approach. LC-MS also revealed that many other proteins secreted by *B. cereus* exist in complexes. Further, a decrease of toxic activity at temperatures ≥60 °C was shown, which was unexpectedly restored at higher temperatures. This could be attributed to a release of Hbl B monomers from tight complexation, resulting in enhanced cell binding. In contrast, Hbl L1 was rather susceptible to heat, while heat treatment of Hbl L2 seemed not to be crucial. Furthermore, Hbl-induced pores had a rather small single-channel conductance of around 200 pS and a probable channel diameter of at least 1 nm on planar lipid bilayers. These were highly instable and had a limited lifetime, and were also slightly cation-selective. Altogether, this study provides astonishing new insights into the complex mechanism of Hbl pore formation, as well as the properties of the pores.

## 1. Introduction

Hemolysin BL (Hbl; [1]) and the non-hemolytic enterotoxin (Nhe; [2]) are the two main enterotoxins responsible for the diarrheal type of food poisoning caused by *Bacillus cereus*. After consumption of contaminated foods and survival of spores in the stomach passage, these enterotoxins are produced by viable cells in the human intestine [3,4]. Both enterotoxin complexes are composed of three individual protein components, namely NheA, B and C; and Hbl L2, L1 and B, respectively. The *nhe* operon can be found in nearly 100%, and the *hbl* operon in about 45–65% of investigated *B. cereus* strains, respectively [5,6,7]. Thus, for a long time detailed investigation of Hbl in *B. cereus* culture supernatants was barely possible, while extensive studies on the pore-forming mode of action of Nhe had been performed [8,9,10,11,12,13,14].

Nevertheless, Hbl was the first of the enterotoxins to be characterized in 1990 [15]. Initially, one binding (B) and one lytic (L) component were identified, later corrected to three components [16]. Hbl was shown to be hemolytic, dermonecrotic and cytotoxic. Moreover, it is active in vascular permeability tests and involved in endophthalmitis and fluid accumulation in rabbit ileal loops [1,15,16,17,18,19,20,21,22]. Recently, its ability to cause inflammasome-mediated mortality in macrophages was demonstrated [23]. Hbl is a pore-forming toxin, which was first shown via osmotic protection assays [24]. All three protein components are necessary for maximum cytotoxicity [1]. Sequential binding in the specific binding order B-L1-L2 is necessary for pore formation [22,25,26]. Pore formation is also determined by the concentration ratio of the individual components, with ratios L2:L1:B = 1:1:10 and 10:1:10 being most effective. Excess of B enhances and excess of L1 decreases the velocity of pore formation, while the amount of L2 seems not to be crucial to complete the pores in the last step [25]. Furthermore, components L1 and B, as well as L1 and L2, form complexes in solution prior to cell binding [26]. It was shown that the presence of L1 enhances binding of B to the target cell surface, which is the most important step in the pore-forming process [25]. Nevertheless, more detailed information about the properties of the Hbl complexes and pores are missing.

The present study describes the size, composition and functionality of the Hbl complexes. Furthermore, it provides new insights into the heat stability and lability of the protein components. Furthermore, basic electrophysiological properties of the Hbl pores are characterized for the first time.

## 2. Results

### 2.1. Size and Composition of the Hbl Complexes

**Figure 1:**
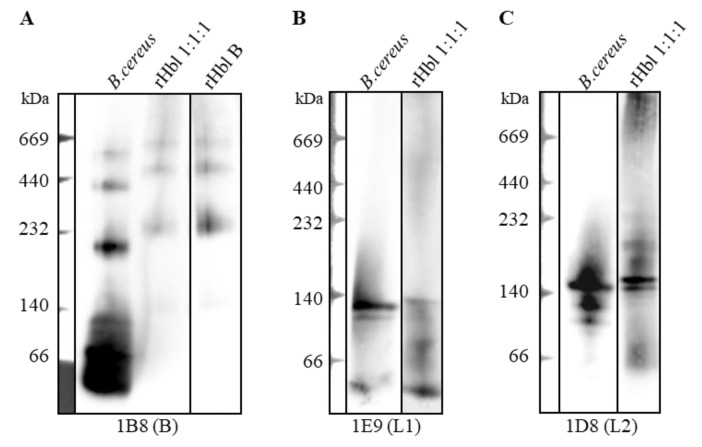
Blue Native PAGE and Western blotting of *B. cereus* F837/76 ∆*nheABC* supernatant (30-fold concentrated) and rHbl (10 pmol/µL each). Note: 1:1:1 = concentration ratio of rHbl B/L1/L2. (**A**) Hbl-B-specific mAb 1B8 was used for detection. (**B**) Hbl-L1-specific mAb 1E9 was used for detection. (**C**) Hbl-L2-specific mAb 1D8 was used for detection.

In a previous study using monoclonal antibody (mAb)-based enzyme immunoassays (EIAs), it was shown that the Hbl components L1 and B, as well as L1 and L2, form complexes in solution [26]. The size, composition and further properties of those complexes remained unclear. To elucidate this point, a supernatant of *B. cereus* strain F837/76 ∆*nheABC* (lacking the *nhe* operon) and rHbl proteins were applied to Blue Native PAGE (polyacrylamide gel electrophoresis), and separated complexes were detected by Western blotting. The use of Hbl-B-specific mAb 1B8 revealed the formation of distinct complexes of up to approximately 600 kDa for the supernatant, as well as the recombinant toxin. Moreover, applying rHbl B alone showed that the structure and size of these complexes were to a large extent determined by Hbl B (Figure 1A). Nevertheless, Hbl L1 and L2 could also be detected in those complexes, mainly at approximately 140 kDa (Figure 1B,C). These data led to the first conclusion that at least rHbl B is almost completely complexed in solution, while in the *B. cereus* supernatant the same complexes exist, along with free, putatively monomeric Hbl B. Furthermore, the properties of the complexes are defined by Hbl B. Hbl L1 and L2 also exist in those complexes, but either in small amounts, or they might not be properly detected in the native complexes due to steric hindrance of mAb binding.

**Figure 2:**
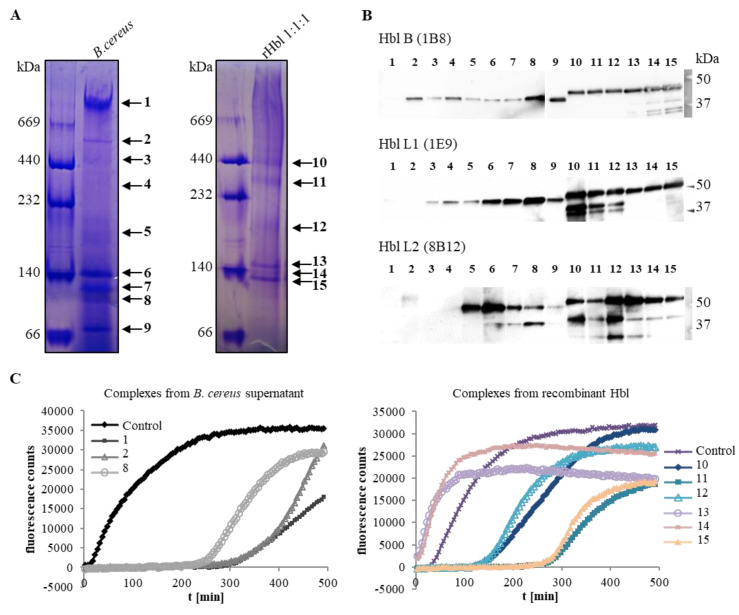
Composition and activity of the Hbl complexes. (**A**) Blue Native PAGE and Coomassie staining of the Hbl complexes from *B. cereus* F837/76 ∆*nheABC* culture supernatant (30-fold concentrated) and rHbl (10 pmol/µL each). Note: 1:1:1 = concentration ratio of rHbl B/L1/L2. Complexes were excised and eluted from the native gel. (**B**) Compositions of the excised complexes as tested by Western blotting with specific mAbs against Hbl B, L1 and L2. (**C**) Pore-forming activities of the complexes after excision and elution as tested via PI influx into Vero cells. Total supernatants of strain F837/76 ∆*nheABC* and rHbl 1:1:1 (37.5 pmol/mL each) were used as controls.

To further determine the composition of the Hbl complexes, native gels were Coomassie-stained and 15 visible bands were excised and eluted from the gel (see Figure 2A and Table 1). Eluted fractions were again applied to Western blotting with specific mAbs against Hbl B, L1 and L2. In contrast to native blotting, Hbl L1 and L2 could be detected in 13 and 11 out of the 15 bands, respectively (Figure 2B and Table 1). The eluted complexes were also tested for their remaining pore-forming activity via propidium iodide (PI) influx into Vero cells (Figure 2C and Table 1). As native elution from the gel slices failed, 0.1% SDS buffer had to be applied, which might have interfered with the activity of the Hbl proteins from the *B. cereus* supernatant. Only bands 1 (700 kDa complex), 2 (600 kDa complex), and 8 (100 kDa complex) showed pore-forming activity after all. On the other hand, the pore-forming activities of all complexes formed from rHbl were recovered. While bands 13 (160 kDa complex) and 14 (140 kDa complex) caused extremely fast PI influx, pore formation occurred more slowly for bands 10 (440 kDa complex) and 12 (200 kDa complex), and was clearly delayed for bands 11 (300 kDa complex) and 15 (120 kDa complex) (Figure 2C and Table 1). Thus, no correlation between the pore forming activity and the size of the complexes could be observed. It has been demonstrated that the ratio of the three single components is essential for this process—the maximum pore forming activity appears at an Hbl L2/L1/B ratio of 1:1:10 [25]—which could not yet be determined for the individual complexes.

Additionally, eight selected bands were investigated via LC-MS (Table 1) to (**i**) verify the presence of Hbl B, L1 and L2 in the complexes; (**ii**) exclude contaminations in the rHbl complexes; and (**iii**) determine further secreted proteins in the *B. cereus* F837/76 ∆*nheABC* supernatant. The results of LC-MS analyses are summarized in Table S1. Beyond our previous findings, Hbl B, L1 and L2 were detected in all analyzed complexes, presumably by circumventing the (steric) limitations of the mAb detection methods. In the investigated rHbl complexes (440, 300 and 200 kDa), Hbl B, L1 and L2 were detected, confirming results of Western blot tests (Figure 2B and Table 1). Furthermore, in the five investigated complexes obtained from the *B. cereus* supernatant, all three Hbl components were found (see Table 1 and Table S1). As *B. cereus* is known to secrete a large number of proteins in the extracellular space, it was not surprising that these were also detected in the present LC-MS approach and that their number increased with decreasing size of the investigated Hbl complex. In particular, proteins such as bacillolysin, chitin binding protein, flagellar proteins, hemolysin II, immune inhibitor A, phospholipase C and different proteases (Table S1) are of major interest, as they were previously described or assumed to be involved in virulence. Some of these proteins might form homo- or heterooligomeric complexes on their own, most likely phospholipase C and sphingomyelinase, and are, thus, found at high molecular weight. Whether there is an interaction of these aggregates with the single Hbl components or Hbl complexes in solution can only be speculated at this point.

Beyond these findings, it was generally observed that complexes formed by rHbl were slightly larger than those in the *B. cereus* supernatant. To exclude interference of the recombinant proteins’ strep tags with complex formation, a new system was established for protein overexpression, purification and subsequent cleavage of the tag (Figure S1A,B). The newly generated proteins—before and after cleavage—showed delayed pore formation into Vero cells compared to the original combination (see Figure S1C and also [26]). On the other hand, the complexes resembled those occurring in the natural *B. cereus* supernatant (Figure S1D).

### 2.2. Heat Stability of the Hbl Complexes

**Figure 3:**
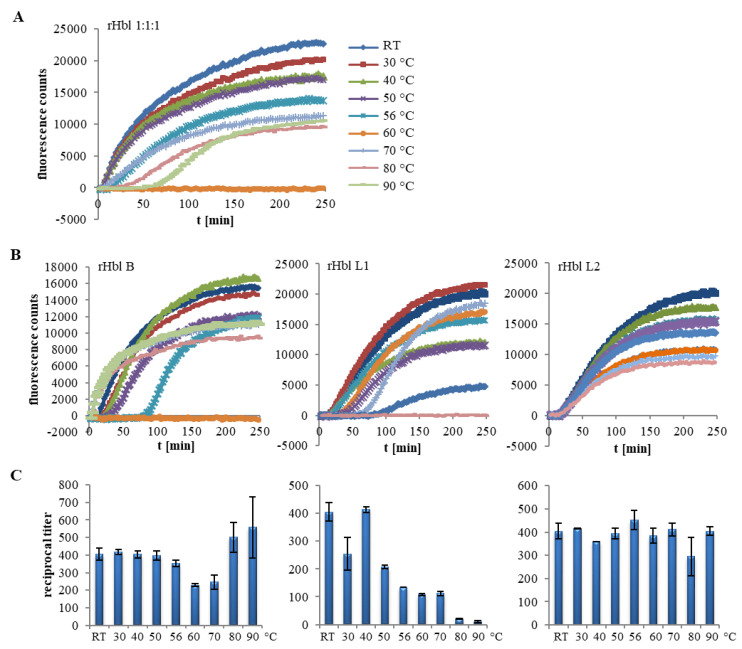
Pore-forming and cytotoxic activity of Hbl depending on heat treatment of the single components. The rHbl components were each set to 1.5 pmol/µL in elution buffer. These were heated individually or as a 1:1:1 mixture for 10 min in 20 µL samples and subsequently applied in the cell culture tests. The end concentrations in the PI influx test equaled 37.5 pmol/mL each. (**A**) PI influx after 10 min treatment of rHbl 1:1:1 with rising temperatures. (**B**) PI influx after incubation of each single rHbl component for 10 min at rising temperatures. Total fluorescence counts differ slightly from those in (**A**). Color scheme as depicted in (**A**). (**C**) Cytotoxic activity determined in WST–1 (water soluble tetrazolium) bioassays after 10 min treatment of each single rHbl component with rising temperatures.

**Figure 4:**
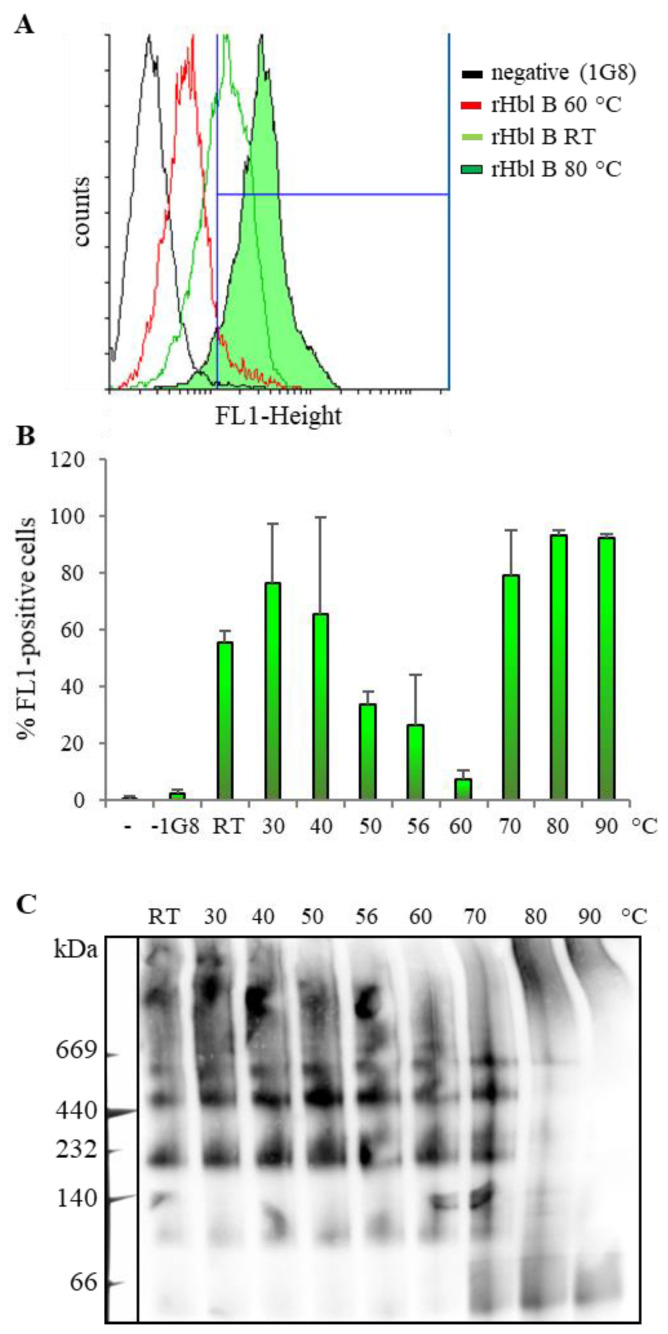
Binding of rHbl B to Vero cells. Protein samples were incubated for 10 min at different temperatures before they were applied for 15 min to Vero cells. Binding was determined via flow cytometry using an Hbl-B-specific mAb 1G8–Alexa 488 conjugate for detection. (**A**) Examples of altered cell binding after heat treatment. (**B**) Means and standard deviations of fluorescence (FL1)-positive cells of two biological replicates, each with three technical replicates. (**C**) Native PAGE and Western blotting of rHbl B after treatment with temperature gradient, using Hbl-B-specific mAb 1B8 for detection.

The first part of this study showed that the Hbl components, especially Hbl B, are highly complexed in solution. However, it is known from earlier studies that Hbl B alone binds to the target cell membrane, representing the initial step of the pore formation [22,25]. It is commonly acknowledged that the *B. cereus* enterotoxins—contrary to the emetic toxin cereulide—are instable towards heat, acid or digestive enzymes [4,27,28]. Contradictorily, it is shown here for the first time that heat treatment can have unexpected effects on the complex formation and pore-forming activity of Hbl. The results of heat treatment experiments are summarized in Figure 3. At first, it was observed that the pore-forming activity of rHbl decreased with increasing incubation temperatures. The PI influx caused by rHbl decreased at 56 °C and was no longer detectable at 60 °C. Unexpectedly, an adjacent increase of PI influx was observed from 70 °C upwards (Figure 3A). The results were similar when the experiment was repeated with *B. cereus* culture supernatant. Subsequently, the pore-forming (Figure 3B) and cytotoxic (Figure 3C) activities of Hbl were tested after treatment of each single component with the temperature gradient to clarify which component was most affected. While both activities decreased when rHbl L1 was treated with increased temperatures, heat treatment seemed not to be crucial for rHbl L2. Whether this is due to the heat stability of L2 or to the fact that only small amounts of L2 are needed for completion of the pores [25] remains to be elucidated. Most interestingly, when different temperatures were applied to rHbl B, pore formation and cytotoxicity decreased at 56 and 60 °C, while PI influx and cytotoxicity titers rose from 70 °C upwards, even surpassing non-treated conditions (Figure 3B,C). Thus, treatment of Hbl B with high temperatures seemed to enhance Hbl activity. To investigate this observation in detail, cell binding of heat-treated rHbl B was determined via flow cytometry. Indeed, heat treatment of rHbl B first resulted in a decrease of cell binding, with its minimum (7% FL1-positive cells) being observed at 60 °C, but from 70 °C upwards, the cell binding ability was increased (up to 93% FL1-positive cells, Figure 4A,B). These findings were consistent with a shift of the Hbl B complexes. While stable complexes were detected on native gels up until 60 °C, an accumulation of bands below 60 kDa, presumably rHbl B monomers, was observed from 70 °C upwards, along with the disappearance of larger complexes (Figure 4C). These findings led to the conclusion that heat treatment might indeed strengthen Hbl activity due to enhanced cell binding by Hbl B monomers released from tight complexation.

### 2.3. Biophysical Characterization of the Hbl Pores

#### 2.3.1. Measurements of the Single-Channel Conductance

While the current knowledge about heat stability or lability of the *B. cereus* enterotoxins might have to be reconsidered, the basic mechanism underlying Hbl pore formation seems to be elucidated. Although for an extended time different models existed [24,29], it is now clear that only sequential binding in the order B-L1-L2 leads to pore formation and cytotoxicity [22,25]. Moreover, binding of Hbl B to the target cell surface is enhanced when the monomers are less tightly stuck to each other, which is obtained (**i**) in the presence of Hbl L1 [25] and (**ii**) after heat treatment (this study). In the present study, *B. cereus* F837/76 ∆*nheABC* supernatants were further used for the biophysical characterization of Hbl pores on planar lipid bilayers. It is worth mentioning that we were not interested in the pores formed by a specific Hbl complex, but rather in the general description of the properties of the pores shaped by Hbl from a *B. cereus* supernatant, which has not been investigated so far.

**Figure 5:**
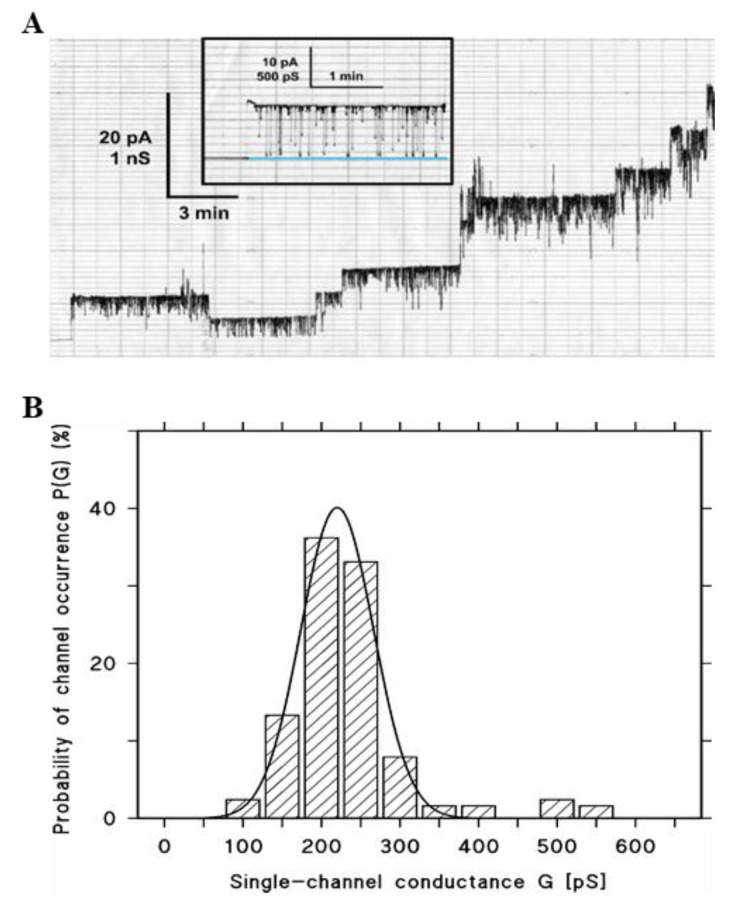
Results of single-channel measurements with supernatant of *B. cereus* F837/76 ∆*nheABC*. (**A**) Single-channel recording of a DiPhPC–n-decane membrane after the addition of about 100 µL supernatant of *B. cereus* F837/76 ∆*nheABC* to one or both sides of the black lipid bilayer (volume 5 mL). The addition of the supernatant resulted in rapid occurrence of single current steps, which immediately showed certain rapid flickering events (see inset for current flickers in 3 M KCl, 10 mM MES, pH6). In addition, the channels had a limited lifetime of about 5 min. The aqueous phase also contained 1 M KCl, 10 mM MES, pH 6. The applied voltage was 20 mV and the temperature was 20 °C. It has to be noted that the rapid flickers were not taken into account for the derivation of the single-channel conductance of the Hbl pores. (**B**) Histogram of the channels formed by the *B. cereus* F837/76 ∆*nheABC* supernatant in DiPhPC–n-decane lipid bilayer membranes using 1 M KCl, 10 mM MES, pH 6 as the electrolyte. The solid line represents a fit of the histogram with a Gaussian distribution. The maximum of the distribution is at a probability for channel conductance of 40 ± 2% and the mean value of the conductance is 219 ± 46 pS, giving a total of 127 single events taken from eight individual membranes, V_m_ = 20 mV, T = 20 °C each.

The first experiments showed that Hbl from the supernatant formed flickering pores with limited lifetimes and an average single-channel conductance of around 200 pS. One representative single-channel recording is depicted in Figure 5A. After opening of an Hbl channel, it started to flicker, however the channel conductance did not reach the baseline during flickering. Closing of the channel occurred only when the residual conductance during flickering also closed. Similar results were obtained when 1% asolectin dissolved in n-decane was used for membrane formation instead of diphytanoyl phosphatidylcholine (DiPhPC), or when cholesterol was added in a 1:2 (cholesterol/DiPhPC) molar ratio. Thus, the type of lipid seemed not to be crucial for Hbl pore formation. The histograms of the single-channel distributions were fairly homogeneous, despite the limited channel lifetime and the rapid flickering of the Hbl pores (see Figure 5B). The average single-channel conductance was 219 ± 46 pS in 1 M KCl, 10 mM MES (2-(*N*-morpholino)ethanesulfonic acid), pH 6. This result indicates that the Hbl pores adapted a defined structure. The flickering of the Hbl pores was obviously not directly related with the closing events, but it cannot be excluded that the channel flicker represents some prerequisite for channel closure. This could be derived from the current recordings shown in Figure 5A and similar measurements of other salts and concentrations.

#### 2.3.2. The Single-Channel Conductance is a Linear Function of the Bulk Aqueous Conductance

To draw a more complete view of the channel properties, single-channel measurements using different salts and concentrations were also performed. The results of these experiments are shown in Table 2. The replacement of chloride by acetate decreased the single-channel conductance by about 20%, whereas the replacement of potassium ions by lithium ones resulted in a more substantial drop by 40%. This indicates that the Hbl pore could be cation selective. The data of Table 2 also contain the concentration dependence of its conductance. The single-channel conductance was in the range between 0.1 M KCl and 3 M KCl an approximately linear function of the bulk aqueous salt concentration. This indicates that the Hbl pore does not contain a hot spot of charges or a binding site for ions, as both conditions would lead to strong deviations from the observed linearity [30,31].

#### 2.3.3. Ionic Selectivity of the Hbl Pores

To directly measure the ion selectivity of the Hbl pores and to confirm the results of the single-channel measurements, zero-current membrane potential experiments were performed in the presence of salt gradients. These experiments were conducted by reconstituting around 50 to 100 Hbl pores in a DiPhPC–n-decane bilayer formed in 0.1 M salt solution. The salt concentration was subsequently increased on one side of the bilayer by adding 3 M salt solution in defined steps until a 0.5 M salt concentration was reached at this side followed by the measurement of the zero-current membrane potential. The results of these experiments are given in Table 3. For all three salts employed in this approach—KCl, K-acetate and LiCl—positive potentials were measured at the more diluted side of the membrane, indicating preferential movement of the cations through the Hbl pores. Analysis of the potentials using the Goldman–Hodgkin–Katz equation verified the cation selectivity, as the ratio of cation permeability P_cation_ divided through the anion permeability P_anion_ was in all cases greater than 1, but did not reach high numbers (see Table 3). Thus, the Hbl pores are cation-selective without being highly selective, similar to the phosphate-specific outer membrane porin OprP of *Pseudomonas aeruginosa* [30] or the channel formed by *Clostridium perfringens* enterotoxin (CPE) [32].

#### 2.3.4. Estimations of the Size of the Hbl Pores

Measurements of the single-channel conductance are not suited to give precise information on the diameter of ion channels, because ion transport through channels depends largely on potential energy barriers inside the channels and not only on their size. Krasilnikov and colleagues proposed a method for the estimation of channel sizes [34,35,36,37]. This method is based on the addition of nonelectrolytes (NEs) to the aqueous salt solutions, which results in an increase of their viscosity and a decrease of the bulk aqueous conductivity. As long as the NEs can enter the channels, they also reduce the single-channel conductance because of the increased viscosity on their inside. When they are too bulky and cannot enter the channels or only a part of them, the single-channel conductance increases up to its value without NEs.

Here, NEs were added at a concentration of 20% (*w/v*) to the aqueous 1 M KCl solution and single-channel experiments with DiPhPC–n-decane membranes were performed. The results of these measurements are listed in Table 4, together with the molecular masses of the NEs, their radii and the conductivity. The latter was approximately 50% of the conductivity in the absence of NEs, indicating an increased viscosity of the NEs-KCl solutions (Table 4). The results suggest that ethylene glycol (M_r_ = 62 g/mol), glycerol (M_r_ = 92 g/mol), sorbitol (M_r_ = 182 g/mol) and maltose (Mr = 360 g/mol) can indeed enter the Hbl pore without any noticeable limitations. This means that the Hbl pore has at least a diameter of 1 nm.

However, the sizing of the Hbl pore using PEGs (polyethylene glycol) as NEs of different molecular masses also demonstrated the limitations of this method. Further sizing was not possible due to strange results of the single-channel conductance measurements. Starting with arabinose (M_r_ = 150 g/mol) and for all PEGs up to M_r_ 1000 g/mol, the single-channel conductance considerably dropped down to values of around 20 to 40 pS, which presumably had nothing to do with the size of the Hbl pore. Several different explanations exist: The pore could have been plugged due to accumulation of PEGs inside the channel, as was observed for the outer membrane p66 channel of *Borrelia burgdorferi* [38]. The NEs could also interfere with the insertion of the Hbl subunits into the membranes or with their assembly. Both effects could lead to truncated pores with smaller single-channel conductance. To get some insight into the processes responsible for the small single-channel conductance in the presence of 20% NEs in the aqueous phase, a much smaller NE concentration of 3.7% for PEG400, PEG600 and PEG1000 was tested. At this concentration, no problem with pore formation occurred. The NE concentrations were subsequently increased to 5.3%, then 6.9% and 8.3%. At the highest concentration, only truncated channels with a conductance similar to that shown in Table 4 were observed. Control experiments with 3.7%, 5.3%, 6.9% and 8.3% maltose did not indicate any irregularities similar to the experiments with 20% maltose. These results demonstrate that the PEGs interfere with the Hbl pores or their subunits in a way that is not yet understood. Interestingly, similar observations were made in concomitantly performed osmotic protection experiments. Jumbled, incomplete inhibition of the hemolytic activity of both the *B. cereus* supernatant and rHbl by different NEs was experienced, which was partly non-reproducible. This points to a rather unspecific interaction of the NEs with the Hbl proteins. As an example, Figure S2A shows hemolysis of the *B. cereus* supernatant (applied as serial dilution) and the incomplete inhibition by all tested NEs. On the other hand, inhibition of rHbl activity by melezitose and PEG 1500 was reproducible and increased with rising concentrations (see Figure S2B), which might, however, point to a pore diameter of approximately 1–2 nm.

#### 2.3.5. Differences of the Hbl Pores from Culture Supernatant and rHbl Proteins

**Figure 6:**
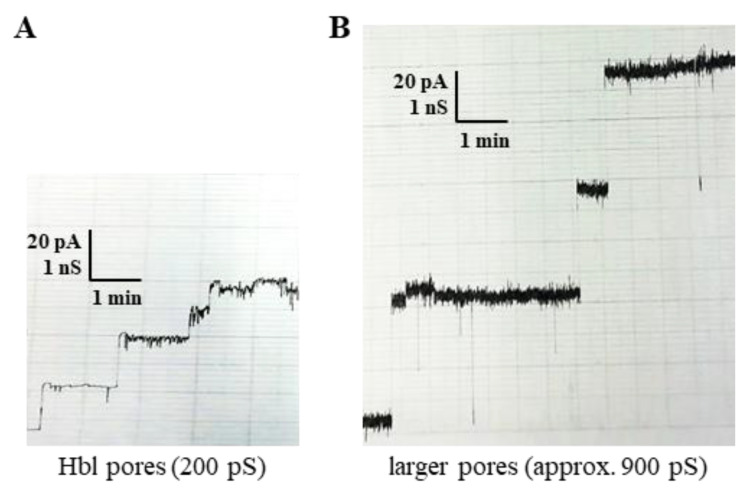
Results of planar lipid bilayer experiments after immunochromatographic removal of Hbl B and L2 from the *B. cereus* supernatant. Experiments were conducted as explained in Figure 5. (**A**) Single-channel recording of a DiPhPC–n-decane membrane infrequently resulted in Hbl pores. (**B**) Very rarely, larger pores with approximately 900 pS conductance were also detected.

We were further interested in the effects of the *B. cereus* supernatant on the lipid bilayers, when the amount of Hbl toxin was minimized. For this, the Hbl components B and L2 were subsequently subtracted from the supernatant via immunoaffinity chromatography. Mostly Hbl L1, as well as traces of L2 and B, remained in the columns’ flow-through. As expected, almost no pore formation was detectable. Only sporadically did the above-described Hbl pores occur (Figure 6A). Even rarer were larger pores with an average single-channel conductance of 900 pS, whose identity remains to be elucidated (Figure 6B).

**Figure 7:**
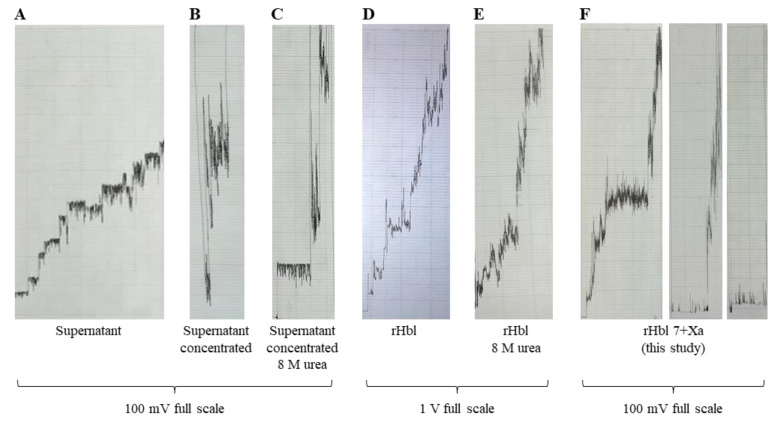
Results of planar lipid bilayer experiments with *B. cereus* F837/76 ∆*nheABC* supernatant, as well as rHbl proteins. (**A**) Small Hbl pores of *B. cereus* F837/76 ∆*nheABC* supernatant for comparison (see also Figure 5A and Figure 6A). (**B**) Lesions resulting from 30-fold concentration of the supernatant. (**C**) Reduction of the lesions of 30-fold-concentrated *B. cereus* F837/76 ∆*nheABC* supernatant by the use of 8 M urea. (**D**) Occurrence of large lesions with rHbl proteins 5 + B, 3 + L1, 5 + L2 (each 1.5 pmol/µl, mixed 1:1:1, [26]). (**E**) No effect of the use of rHbl proteins 5 + B, 3 + L1, 5 + L2 (each 1.5 pmol/µl, mixed 1:1:1, [26]) in 8 M urea. (**F**) Reduction of the large lesions of rHbl by the use of the newly developed 7 + rHbl proteins without strep tag.

When a combination of any two rHbl components was used in these assays, no pore-forming activity was observed, which confirmed earlier observations [25]. However, when all three originally purified rHbl components [26] were applied instead of the *B. cereus* supernatant, rather large lesions appeared instead of the small 200 pS pores (see Figure 7A,D for comparison). Slightly larger lesions could be produced using 30-fold concentrations of the *B. cereus* supernatant (Figure 7B), which was partly undone by the addition of 8 M urea (Figure 7C). However, 8 M urea had no effect on the large lesions established by rHbl (Figure 7E). Only when the rHbl components without tag (generated in this study, refer to Figure S1) were applied were smaller lesions and occasionally the 200 pS Hbl pores observed (Figure 7F). This led to the conclusion that although no effect has been observed on target cells so far, the strep tag of the rHbl proteins might interfere with pore formation on planar lipid bilayers.

## 3. Discussion

The present study addressed the properties of hemolysin BL complexes in solution, as well as the biophysical characteristics of its pores on lipid bilayers. Distinct complexes of up to 600 kDa were identified on native PAGE in *B. cereus* culture supernatant and for rHbl proteins (see Figure 1). In subsequent Western blots and LC-MS analyses, all three Hbl components could be identified in each complex. This was not surprising, as a previous study had already shown complex formation between Hbl L1 and B, as well as between L1 and L2 via EIAs. This was confirmed by surface plasmon resonance, with K_D_ values of 4.7 × 10^−7^ M and 1.5 × 10^−7^ M, respectively. Here, a weaker binding between Hbl B and L2 with a K_D_ value of 3.4 × 10^−6^ M was also detected, which could not be observed using the antibody-based system [26]. The latter detection system might be limited due to steric hindrance of mAb binding to their target proteins—especially in the case of strong complexation, or when only one or a few molecules are hidden in the complex. The present results let us propose this at least for Hbl L2. It has already been suggested that concerning the non-hemolytic enterotoxins, only one molecule NheC is probably complexed with up to 15 molecules NheB [14]. Furthermore, the present experiments let to the conclusion that the size and properties of the complexes are mostly defined by Hbl B. The current findings regarding the complexes are a further step towards complete elucidation of the mode of pore formation by Hbl. It is known that the three components bind sequentially to the target cell surface in the required order, B-L1-L2 [22,25]. Although Hbl B is able to bind the cell surface on its own, binding is enhanced in the presence of L1 [25], which underlines the importance of preceding complex formation [26]. Nevertheless, a sufficient amount of free Hbl B is also required [25], which could be detected in the supernatant of strain F837/76 ∆*nheABC* (see Figure 1A). After this, free Hbl L1 can rapidly attach to cell-bound Hbl B [22,25]. The optimal concentration ratio for maximum pore formation is Hbl L2:L1:B = 1:1:10. The last step is the immediate attachment of Hbl L2, presumably followed by conformational changes enabling the manifestation of the full pore.

The complexation of Hbl B might also explain why up to 15-fold lower concentrations of this protein are routinely found in different *B. cereus* culture supernatants compared to Hbl L1 or L2 when applying EIAs. While this was so far explained by the transcription of the *hblCDA* operon as one polycistronic mRNA [42], it might depend on the inaccessibility of the Hbl B protein in the complexes. Thus, more Hbl B protein is present in *B. cereus* culture supernatants, as previously assumed. As it was already shown that Hbl B is the crucial component for pore formation and cytotoxic activity of the complex [25], this must also be taken into account regarding the development of antibody-based detection systems, as well as risk evaluation of *B. cereus*.

Furthermore, the present study showed that Hbl B monomers can be released from the complexes by heat treatment, resulting in intensified cell binding, and thus enhanced pore formation and cytotoxicity (see Figure 3 and Figure 4). Some parallels can be drawn to Nhe, where a defined level of NheB-C complexes, as well as a sufficient amount of free NheB protein, is necessary for cell binding and cytotoxicity [11]. Complex formation of either NheB and C or Hbl B, L1 and L2 seems to be a “balanced process, necessary to induce, but also able to limit” pore-forming, and thus cytotoxic activity. At least for Hbl, this seems to depend to a large extent also on extrinsic factors such as heat treatment. A similar suggestion has been made in an earlier study, when Hbl was exposed to increasing pressure at different temperatures [28]. A comparable principle is known for oligomeric heat shock proteins, which undergo conformational changes, aggregation, dynamic dissociation or reassociation at temperatures above 60 °C, activating or increasing their chaperone activities [43,44,45,46]. Equally to heat treatment, similar initially inconclusive results were obtained for pore formation when the single Hbl components were exposed to low pH values. Consequently, the general opinion of the “classic” *B. cereus* enterotoxins, which are highly susceptible towards re-heating of contaminated foods or stomach acid, might have to be reconsidered.

The present study also showed for the first time distinct characteristics of the Hbl pores. Application of supernatant of *B. cereus* F837/76 ∆*nheABC* to planar lipid bilayers resulted in instable, “flickering”, slightly cation-selective, presumably 1–2 nm diameter pores with a single-channel conductance of about 200 pS and a limited lifetime (see Figure 5, Figure 6 and Figure 7, as well as Table 2, Table 3 and Table 4). Application of any two rHbl components in the bilayer experiments had no effect. Hence, in contrast to all parallels between Nhe and Hbl regarding complex formation, a different image of their pores appeared. While NheB and C alone are able to form permeable “pro-pores” with a conductivity of approximately 870 pS and a diameter of 2 nm [11,14], these seem not to exist for Hbl. Thus, the complete Nhe pore [9,47] is assumed to be a lot larger than the Hbl pore, which might be one explanation for their different impacts on target cells. In an older study, it was already observed that the Hbl pores were formed faster in the target cell membrane, but that Nhe eventually caused more damage to the cells [20]. Due to structural similarities, *B. cereus* Nhe and Hbl belong to the ClyA superfamily of α-helical pore forming toxins [10,29]. ClyA or SheA (silent haemolysin A) or HlyE (haemolysin E) forms 3 nm, homo-dodecameric pores by oligomerization and formation of a ring-shaped structure after cholesterol binding on the cell surface [48,49,50]. What the three toxins have in common is their protein structure, consisting of a tail (α-helices) and a head domain (β-tongue) [10,51], as well as the moderate selectiveness for cations of the corresponding pores ([10,49], as well as this study). ClyA also forms complexes in solution, which are linear oligomers of different sizes. In contrast to Nhe and Hbl, these are homo-oligomeric [51,52]. Similar to Nhe, ClyA can also permeabilize membranes before complete pore formation [53]. Moreover, the complete pores of ClyA and Nhe are highly stable, which is another significant difference to Hbl.

In the present study, Hbl from natural *B. cereus* supernatant and rHbl proteins acted differently on the lipid bilayers, contrary to their application on target cells [25,26]. First, rHbl caused large, rather unspecific lesions due to an accumulation of the toxin (either an accumulation of pores on the membranes, or more likely, large, accumulated complexes, which were inserted into the membranes). For the supernatant, this accumulation was simulated by 30-fold concentration of the sample, and could be reversed by the use of 8 M urea. Urea could not reverse the rHbl lesions. Consequently, it is rather possible that the additional strep tags and linker sequences of the rHbl proteins interfered with the highly sensitive bilayer experiments. This could only be improved when newly generated rHbl proteins lacking the tag were used (see Figure 7). Impairment of the bilayer experiments by protein tags has previously been observed for *Corynebacterium jeikeium* porin PorACj [54]. Furthermore, the formation of large lesions can also be determined by extrinsic factors such as pH. As an example, *Clostridium difficile* toxin B showed small channel-forming activity at pH 6. After lowering the pH to 5, the membrane conductance increased due to the formation of many channels [55]. Interestingly, when large parts of Hbl components B and L2 were removed from the *B. cereus* supernatant via immunoaffinity chromatography, larger pores with an average single-channel conductance of 900 pS were infrequently observed next to some residual Hbl pores (see Figure 6). Beyond the enterotoxins, *B. cereus* is known to produce a variety of secreted proteins, especially virulence-related factors [56,57]. A number of these toxins, degradative enzymes, adhesins or flagellar components [56] were also identified in the *B. cereus* supernatant in the present study (see Table S1). As one example, hemolysin II is an oligomeric ß-barrel pore-forming toxin, such as *Staphylococcus aureus* α-toxin, *C. perfringens* ß-toxin or *B. cereus* cytotoxin K (CytK) [58,59,60]. HlyII has been shown to also form anion-selective pores on lipid bilayers with an inner diameter of 1.5–2 nm and an outer diameter of 6–8 nm, as well as a most probable single-channel conductance of 342 pS [58]. Additionally, thiol-activated cytolysins (Table S1) have attracted attention, as they are to some extent related to cholesterol-dependent cytolysins (CDCs) such as *C. perfringens* perfringolysin O, *Paenibacillus alvei* alveolysin or streptolysin O of different streptococci [60].

## 4. Conclusions

Altogether, although it must not be neglected that further pore-forming proteins are secreted by *B. cereus*, the tripartite enterotoxins Nhe and Hbl are the main contributors to pore formation and cytotoxicity. The present study underlined once more that these two toxin complexes exhibit different effects to lipid bilayers, and thus to their target cells, despite their sequential and structural homologies. While the smaller Hbl pores are formed in the cell membrane immediately after application, the larger, complete Nhe pores eventually cause more damage to the cells. It might be the synergy of these two enterotoxins that makes diarrheal *B. cereus* food infections even more effective.

## 5. Materials and Methods

### 5.1. Bacterial Strains and Culture Conditions

In this study, the *B. cereus* strain F837/76 (DSM 4222) ∆*nheABC* [26] was used. The strain was grown in CGY medium with 1% glucose for 6 h at 32 °C with shaking to obtain toxin-rich culture supernatant. Subsequently, the sample was centrifuged (4000× *g* at 4 °C for 20 min), and after addition of 1 mM EDTA, filtered through a 0.2 µm sterile filter. *E. coli* strain BL21 (DE3) grown in LB medium containing 100 µg/mL ampicillin was used for overexpression of recombinant (r) Hbl proteins (see [26]).

### 5.2. Cell Line and Culture Conditions

Vero cells were obtained from European Collection of Cell Cultures (ECACC) and cultured in 80 cm^2^ culture flasks in a humidified incubator at 37 °C and 7% CO_2_ in MEM (Minimum Essential Medium) Earle’s medium (Merck, Darmstadt, Germany) plus supplements, as recommended by the supplier.

### 5.3. Immunoaffinity Chromatography

To remove Hbl components B and L2 from the supernatant of strain F837/76 ∆*nheABC*, immunoaffinity chromatography columns were used with Hbl-B-specific monoclonal antibody (mAb) 1B8 or Hbl-L2-specific mAb 1A12 [7,61] coupled to CNBr-activated sepharose 4B. After washing of the column with 20 mL PBS, 20 mL culture supernatant was applied. After another washing step with 20 mL PBS, Hbl B or L2 were eluted by adding 10 mL glycine–HCl solution (pH 2.5). The eluate was immediately neutralized with 1 M Tris (pH 7.0) and dialyzed against PBS three times overnight at 4 °C.

### 5.4. Enzyme Immunoassays (EIAs)

For the detection of Hbl components, indirect and sandwich enzyme immunoassays were performed as described before [6,61,62,63]. Established mAbs were used: 10 µg/mL 1A12/1H9-HRP 1:2000 for Hbl L2, 1 µg/mL 1E9 for Hbl L1 and 2 µg/mL 1B8 for Hbl B [7,26,61].

### 5.5. Production of Purified, Recombinant (r) Hbl Components

Hbl proteins (rHbl B and L2 with N-terminal and rHbl L1 with C-terminal strep tag) were overexpressed in *E. coli* BL21 (DE3) and purified as described earlier [26]. In the present study, a system for separation of the tag was established. The genes *hblC* (L2), *hblD* (L1) and *hblA* (B) were amplified by PCR using pASK-IBA5+hblC, pASK-IBA3+hblD and pASK-IBA5 + hblA [26] as templates, as well as the primer pairs L1_7+_fw (ATATCCGCGGTGCACAAGAAACGACCG) and L1_7+_rev (ATATGTCGACCTACTCCTGTTTAAAAGCAATATC), L2_7+_fw (ATATCCGCGGTCAAGCAGAAACTCAACAAGA) and L2_7+_rev (ATATGTCGACTCAAAATTTATACACTTGTTCTTC), and B_7+_fw (ATATCCGCGGTGCAAGTGAAATTGAACAAAC) and B_7+_rev (ATATGTCGACCTATTTTTGTGGAGTAACAGTTTC). Using the enzymes SacII and SalI (New England Biolabs, Frankfurt, Germany; restriction sites underlined in the primer sequences), the three genes lacking the sequences for the N-terminal signal peptides for secretion [64,65] were cloned into pASK-IBA7+ (IBA Lifesciences, Göttingen, Germany). The constructs were sequenced using the primers pASK-IBA-seq-fw (CACTCCCTATCAGTGATAG) and pASK-IBA-seq-rev (GCACAAT GTGCGCCAT). Proteins were again overexpressed in *E. coli* BL21 (DE3) and purified via their N-terminal strep tag as described earlier [26].

### 5.6. Factor Xa Digestion

A special feature of the pASK-IBA7+ vector system is the introduction of the factor Xa binding site at the four amino acid sequence “IEGR” close to the strep tag at the protein’s N-terminus. To remove the tag, 1.4 µL of factor Xa protease (New England Biolabs, Frankfurt, Germany) was added to 1.5 pmol/µL of the purified rHbl 7 + proteins in 100 µL buffer (100 mM Tris, 300 mM NaCl, pH 8). The mixture was incubated for 6 h at room temperature and subsequently stored at −20 °C.

### 5.7. Native PAGE

For separation on native gels, the supernatant of strain F837/76 ∆*nheABC* [26] was washed with PBS 1:5 and concentrated 30-fold using Amicon Ultra–4 30 kDa centrifugal filters (Millipore; Merck, Darmstadt, Germany) according to the instructions of the manufacturer. The purified rHbl components L2, L1 and B [26] were treated equally and concentrated to 10 pmol/µL each. For electrophoresis, the NativePAGE^TM^ Novex^®^ Bis-Tris Gel System (Invitrogen; Thermo Fisher Scientific, Waltham, MA, USA) was used. Each sample (16.25 µL) was mixed with 6.25 µL NativePAGE^TM^ sample buffer (50 mM Bis-Tris, 6 N HCl, 50 mM NaCl, 10% Glycerol, 0.001% Ponceau S, pH 7.2) and 2.5 µL NativePAGE^TM^ 5% Coomassie G–250 sample additive, then subsequently applied to 3–12% Bis-Tris protein gels. A high molecular weight protein standard was added (Amersham HMW Calibration Kit for Native Electrophoresis; GE Healthcare, Munich, Germany). Electrophoresis took place for 2.5 h at 150 V in NativePAGE^TM^ running buffer (50 mM Bis-Tris and 50 mM Tricine). The gel was either stained for 20 min in 0.02% Coomassie R–250 in 30% methanol and 10% acetic acid then destained in 8% acetic acid, or applied to Western blotting.

### 5.8. Liquid Chromatography–Mass Spectrometry (LC-MS)

Bands from Coomassie-stained native gels were excised with a sterile scalpel and transferred to 1.5 mL reaction tubes. Sample preparation and LC-MS measurement took place at Rudolf Virchow Center for Experimental Biomedicine, Würzburg, Germany. The excised bands were destained with 30% acetonitrile in 0.1 M NH_4_HCO_3_ (pH 8), shrunk with 100% acetonitrile, and dried in a vacuum concentrator (Concentrator 5301; Eppendorf, Germany). In-gel digestion was performed with 0.1 µg trypsin per gel band overnight at 37 °C in 0.1 M NH_4_HCO_3_ (pH 8). After removing the supernatant, peptides were extracted from the gel slices with 5% formic acid and extracted peptides were pooled with the supernatant.

NanoLC-MS/MS analyses were performed on an Orbitrap Fusion (Thermo Fisher Scientific, Waltham, MA, USA) equipped with a PicoView Ion Source (New Objective, Woburn, MA, USA) and coupled to an EASY-nLC 1000 (Thermo Fisher Scientific, Waltham, MA, USA). Peptides were loaded on capillary columns (PicoFrit, 30 cm × 150 µm ID; New Objective, Woburn, MA, USA), self-packed with ReproSil-Pur 120 C18-AQ, 1.9 µm (Dr. Maisch GmbH, Germany), and separated with a 30 min linear gradient ranging from 3% to 30% acetonitrile, with 0.1% formic acid and a flow rate of 500 nL/min.

Both MS and MS/MS scans were acquired in the Orbitrap analyzer, with a resolution of 60,000 for MS scans and 7500 for MS/MS scans. HCD fragmentation with 35% normalized collision energy was applied. A top speed, data-dependent MS/MS method with a fixed cycle time of 3 s was used. Dynamic exclusion was applied with a repeat count of 1 and an exclusion duration of 30 s; singly charged precursors were excluded from selection. The minimum signal threshold for precursor selection was set to 50,000. Predictive AGC (automatic gain control) was used, with a target value of 2e5 for MS scans and 5e4 for MS/MS scans. EASY-IC (internal calibration) ion source assembly was used for internal calibration.

A Mascot Distiller 2.7 (Matrix Science Ltd., London, UK) was used for raw data processing and for generating peak lists, essentially with standard settings for the Orbitrap Velos (high/high settings). Mascot Server 2.5 was used for database searching with the following parameters: peptide mass tolerance: 10 ppm; MS/MS mass tolerance: 0.02 Da; enzyme: “trypsin”, with 3 missed cleavage sites allowed; variable modifications: acetyl (protein N-term), Gln → pyroGlu (N-term Q) and oxidation (M). Database searching was performed against UniProt *Bacillus cereus* Reference Proteome database (UP000001417), as well as the sequences of the Hbl proteins of *B. cereus* strain F837/76. Table S2 gives an overview of the LC-MS raw data. The mass spectrometry proteomics data have been deposited to the ProteomeXchange Consortium [66] via the PRIDE [67] partner repository with the dataset identifier PXD021720.

### 5.9. Protein Elution from Native PAGE

Protein complexes were eluted from native PAGE gel slices using Model 422Electro-Eluter (BioRad Laboratories, Feldkirchen, Germany) following the instructions of the manufacturer. In brief, gel slices were added to glass tubes sealed at their lower end with frits and membrane caps. Glass tubes and gel chambers were filled with SDS elution buffer (25 mM Tris-base, 192 mM Glycin, 0.1% SDS). Elution was performed for 4 h at 10 mA per glass tube.

### 5.10. SDS-PAGE, Sypro Staining and Immunoblotting

Western blots using Hbl-specific monoclonal antibodies were applied to (**i**) verify separation of the strep tags from the rHbl proteins, (**ii**) detect the Hbl complexes on native PAGE and (**iii**) detect single Hbl components in the complexes eluted from native PAGE.

For (**i**), SDS-PAGE was performed using a PhastGel gradient (10 to 15%) minigel system (GE Healthcare, Munich, Germany) according to the instructions of the supplier. For (**ii**) and (**iii**), 12% Bis–Tris gels were used in a Criterion Cell (Bio-Rad Laboratories, Feldkirchen, Germany) for 1 h at 200 V. Proteins were blotted to a polyvinylidene fluoride P membrane (Millipore; Merck, Darmstadt, Germany). The membrane was blocked for 1 h in 3% casein–PBS, before 2 µg/mL of the specific mAbs was applied for 1 h at room temperature. Hbl L2: 8B12, 1D8; Hbl L1: 1E9; Hbl B: 1B8 [26,61]; purified rHbl components: StrepMAB Classic (IBA Lifesciences, Göttingen, Germany). After 3 washing steps in PBS with 0.1% Tween 20, rabbit anti-mouse–horseradish peroxidase conjugate (Dako; Merck, Darmstadt, Germany) was applied at 1:2000 dilution in 1% casein–PBS for 1 h. After three further washing steps in PBS with 0.1% Tween 20 and two in PBS, membranes were incubated for 2 min with Super Signal Western Femto Maximum Sensitivity Substrate (Pierce; Thermo Fisher Scientific, Waltham, MA, USA). Chemiluminescence signals were detected on a UVP ChemStudio imager (Analytik Jena, Jena, Germany).

### 5.11. Cytotoxicity Assays

Cytotoxicity assays were performed as previously described [20,61,62]. Briefly, for WST–1 bioassays, serial dilutions of rHbl proteins were placed into 96-well plates (0.1 mL per well). Subsequently, Vero cell suspensions (1 × 10^4^ cells and 0.1 mL per well) were added and incubated for 24 h at 37 °C and 7% CO_2_. Cell viability was determined after addition of WST–1 (Roche diagnostics; Merck, Darmstadt, Germany) and further incubation for 1 h. Optical density values were recorded in a Tecan infinite F50 photometer at 450 nm. Dose–response curves were calculated to obtain 50% lethal concentrations, which are shown as reciprocal titers. Propidium iodide (PI) influx tests were applied to measure pore formation in the membranes of Vero cells. Briefly, 4 × 10^4^ Vero cells were seeded in 200 µL MEM Earle’s medium per well in black 96-well plates and incubated for 24 h at 37 °C and 7% CO_2_. Then, 100 µL medium was removed and 100 µL fresh medium was added, containing 10 µg/mL PI (Sigma-Aldrich; Merck, Darmstadt, Germany) and *B. cereus* culture supernatant (1:40) or rHbl components (37.5 pmol/ml). Fluorescence was measured immediately in a Victor 1420 multilabel counter (Perkin Elmer, Waltham, MA, USA) for 4 h every 2.5 min (excitation: 530 nm; emission: 616 nm; excitation time: 1 s; excitation strength: 20.000). Fluorescence curves of three replicates were calculated using Microsoft Excel.

### 5.12. Flow Cytometry

For flow cytometry analyses, Vero cells were adjusted to 1 × 10^6^ cells in 1 mL EC buffer (140 mM NaCl, 15 mM HEPES, 1 mM MgCl_2_, 1 mM CaCl_2_, 10 mM glucose pH 7.2). Subsequently, pre-heated rHbl B was added to a final concentration of 6.5 pmol/mL. The mixture was incubated for 15 min at 37 °C under moderate agitation before 2 mL 1% BSA–PBS was added and cells were centrifuged for 5 min at 800 rpm. Cells were washed again in 2 mL 1% BSA-PBS. After this, the samples were incubated with 3 µg/mL Hbl-B-specific AlexaFluor^®^ 488 (Invitrogen; Thermo Fisher Scientific, Waltham, MA, USA)-labelled mAb 1G8 for 1 h at 4 °C. After additional washing, samples were resuspended in 500 µL 1% BSA-PBS and measured in a FACS Calibur using CellQuestPro software (BD Bioscience, Heidelberg, Germany). Data were analyzed using Flowing Software version 2.5.1.

### 5.13. Planar Lipid Bilayer Assays

Planar lipid bilayer experiments were performed for the biophysical characterization of the Hbl pores [68]. If not stated otherwise, membranes were obtained from a 1% (*w/v*) solution of diphytanoyl phosphatidylcholine (DiPhPC, Avanti Polar Lipids, Alabaster, AL, USA) dissolved in n-decane (C10) [69]. Membranes were also formed from asolectin (Sigma-Aldrich; Merck, Darmstadt, Germany), or from DiPhPC–cholesterol mixtures (Sigma-Aldrich; Merck, Darmstadt, Germany) at defined molar ratios. Asolectin is a lipid mixture from soybeans that contains about 40% neutral phospholipids [70]. The membranes were formed across circular holes (surface area about 0.4 mm^2^) in a wall separating two aqueous compartments in a Teflon cell at volumes of 5 mL each. The salts used for the preparation of the aqueous salt solutions were analytical grade and were buffered with MES to pH6. The temperature was maintained at 20 °C. Two Ag/AgCl electrodes (Metrohm, Herisau, Switzerland) with salt bridges were immersed into the two aqueous compartments of the Teflon cell and connected in series with a home-made voltage source and a current amplifier (Keithley 427, Cleveland, OH, USA). The amplified signal was detected on an oscilloscope (Owon, Zhangzhou, China) and recorded with a strip chart recorder (Rikadenki, Freiburg, Germany). *B. cereus* culture supernatant or recombinant Hbl proteins were added to both aqueous compartments of the Teflon chamber when the membrane was in the black state. At least 100 single events were recorded for the evaluation of the single-channel conductance of the Hbl pores under the different conditions. The ionic selectivity of the Hbl pores was measured via the zero-current membrane potentials of five-fold salt gradients across membranes containing at least 50 Hbl pores [33]. The potentials were analyzed using the Goldman–Hodgkin–Katz equation. The experiments with non-electrolytes performed to size the Hbl pores were performed essentially as described [14,38].

### 5.14. Hemolysis Assays

Defibrinated sheep blood (Oxoid; Thermo Fisher Scientific, Waltham, MA, USA) was centrifuged for 5 min at RT and 590 g, then washed three times in 150 mM NaCl, 5 mM Tris-HCl, pH 7.2. Subsequently, the blood concentration was set to 2% in the buffer. *B. cereus* supernatant or rHbl was added as a dilution series to 800 µL total volume. Erythrocytes in 800 µL buffer served as negative control, while erythrocytes in 800 µL H_2_O served as positive control for hemolysis. Samples were incubated for 30 min at 37 °C. Afterwards, the optical density at 562 nm was determined in triplicate in a Tecan Infinite F50 photometer. Non-electrolytes were added to the samples at concentrations stated in the results section.

## Figures and Tables

**Table 1:** Properties of the Hbl complexes excised from Blue Native gels. WB: detection of Hbl components in Western blots; LC-MS: detection of Hbl components via additionally performed liquid chromatography mass spectrometry; −: no signal; (+): weak signal; +: clear signal; ++: strong signal; +++: very strong signal; nd: not determined.

Band No	Approximate Size (kDa)	Hbl B WB	Hbl L1 WB	Hbl L2 WB	Pore Formation	Additional LC-MS
*B. cereus* supernatant						
1	700	(+)	−	−	+	+
2	600	+	−	−	+	+
3	440	+	+	−	−	+
4	300	+	+	−	−	nd
5	200	+	+	+	−	nd
6	140	+	+	+	−	nd
7	120	+	+	+	−	+
8	100	+	+	+	++	nd
9	70	+	+	+	−	+
rHbl						
10	440	+	+	+	++	+
11	300	+	+	+	+	+
12	200	+	+	+	++	+
13	160	+	+	+	+++	nd
14	140	+	+	+	+++	nd
15	120	+	+	+	+	nd

**Table 2:** Average single-channel conductance of Hbl pores in different electrolyte solutions. The membranes were formed from DiPhPC–n-decane. The single-channel conductance was measured at 20 mV applied voltage and T = 20 °C. The average single-channel conductance, G (± SD), was calculated from at least 100 single events by using Gaussian Distributions similar to that shown in Figure 5B.

Electrolyte	Concentration (M)	G (pS)
KCl	0.1	24 ± 5.0
	0.3	75 ± 13
	1	219 ± 46
	3	610 ± 58
LiCl	1	133 ± 50
KCH_3_COO (pH 7)	1	180 ± 39

**Table 3:** Zero-current membrane potentials, V_m_, of DiPhPC–n-decane membranes in the presence of supernatant of *B. cereus* F837/76 ∆*nheABC* measured for 5-fold gradients of different salts. V_m_ is defined as the difference between the potential at the diluted side and the potential at the concentrated side. The aqueous salt solutions were buffered with MES and had a pH of 6, if not indicated otherwise; T = 20 °C. The permeability ratios P_cation_/P_anion_ were calculated using the Goldman–Hodgkin–Katz equation as the mean (± SD) of at least three individual experiments [33].

Electrolyte	Permeability Ratios P_cation_/P_anion_	V_m_ (mV)
KCl	3.3	19.1 ± 0.8
LiCl	2.3	13.7 ± 0.5
KCH_3_COO (pH 7)	5.0	25.5 ± 0.6

**Table 4:** Average single-channel conductance of the Hbl pores in the presence of different nonelectrolytes (NEs) in the bath solution. Average single-channel conductance G and its standard deviation SD were calculated from Gaussian distributions of the conductance steps. The aqueous phase contained 1 M KCl and the corresponding NE at a concentration of 20% (*w/v*). V_m_ = 50 mV; T = 20 °C. Mr = molecular mass; r = hydrodynamic radius; Mr and r of the NEs were taken from previous publications [39,40,41]; χ = conductivity of the aqueous solutions [38].

NE	Mr (g/mol)	r (nm)	G ± SD (pS)	χ (mS cm^−1^)
None	-	-	219 ± 46	110.3
Ethylene glycol	0062	0.26	125 ± 22	157.2
Glycerol	0092	0.31	132 ± 25	149.1
Arabinose	0150	0.34	42 ± 5.3	163.7
Sorbitol	0182	0.39	148 ± 24	157.8
Maltose	0360	0.50	121 ± 17	173.8
PEG 200	0200	0.50	40 ± 6.5	146.1
PEG 400	0400	0.70	36 ± 4.8	146.4
PEG 600	0600	0.80	25 ± 4.2	154.1
PEG 1000	1000	0.94	22 ± 5.5	149.5

## Supplementary Materials

The following are available online at https://www.mdpi.com/2072-6651/12/11/672/s1: Figure S1: Properties of the rHbl components overexpressed using the pASK IBA 7 + system. Figure S2: Results of hemolysis assays with defibrinated sheep blood. Table S1: Results of LC-MS analyses. Table S2: Overview of LC-MS experiments.

## References

[1] Beecher D.J., Schoeni J.L., Wong A.C. (1995). Enterotoxic activity of hemolysin BL from *Bacillus cereus*. Infect. Immun..

[2] Lund T., Granum P.E. (1996). Characterisation of a non-haemolytic enterotoxin complex from *Bacillus cereus* isolated after a foodborne outbreak. FEMS Microbiol. Lett..

[3] Jessberger N., Kranzler M., Da Riol C., Schwenk V., Buchacher T., Dietrich R., Ehling-Schulz M., Märtlbauer E. (2019). Assessing the Toxic Potential of Enteropathogenic *Bacillus cereus*. Food Microbiol..

[4] Stenfors Arnesen L.P., Fagerlund A., Granum P.E. (2008). From soil to gut: *Bacillus cereus* and its food poisoning toxins. FEMS Microbiol. Rev..

[5] Guinebretiére M.H., Broussolle V., Nguyen-The C. (2002). Enterotoxigenic profiles of food-poisoning and food-borne *Bacillus cereus* strains. J. Clin. Microbiol..

[6] Moravek M., Dietrich R., Bürk C., Broussolle V., Guinebretiére M.H., Granum P.E., Nguyen-The C., Märtlbauer E. (2006). Determination of the toxic potential of *Bacillus cereus* isolates by quantitative enterotoxin analyses. FEMS Microbiol. Lett..

[7] Wehrle E., Moravek M., Dietrich R., Bürk C., Didier A., Märtlbauer E. (2009). Comparison of multiplex PCR, enzyme immunoassay and cell culture methods for the detection of enterotoxinogenic *Bacillus cereus*. J. Microbiol. Methods.

[8] Didier A., Dietrich R., Gruber S., Bock S., Moravek M., Nakamura T., Lindbäck T., Granum P.E., Märtlbauer E. (2012). Monoclonal antibodies neutralize *Bacillus cereus* Nhe enterotoxin by inhibiting ordered binding of its three exoprotein components. Infect. Immun..

[9] Didier A., Dietrich R., Märtlbauer E. (2016). Antibody Binding Studies Reveal Conformational Flexibility of the *Bacillus cereus* Non-Hemolytic Enterotoxin (Nhe) A-Component. PLoS ONE.

[10] Fagerlund A., Lindbäck T., Storset A.K., Granum P.E., Hardy S.P. (2008). *Bacillus cereus* Nhe is a pore-forming toxin with structural and functional properties similar to the ClyA (HlyE, SheA) family of haemolysins, able to induce osmotic lysis in epithelia. Microbiology.

[11] Heilkenbrinker U., Dietrich R., Didier A., Zhu K., Lindbäck T., Granum P.E., Märtlbauer E. (2013). Complex formation between NheB and NheC is necessary to induce cytotoxic activity by the three-component *Bacillus cereus* Nhe enterotoxin. PLoS ONE.

[12] Lindbäck T., Fagerlund A., Rodland M.S., Granum P.E. (2004). Characterization of the *Bacillus cereus* Nhe enterotoxin. Microbiology.

[13] Lindbäck T., Hardy S.P., Dietrich R., Sodring M., Didier A., Moravek M., Fagerlund A., Bock S., Nielsen C., Casteel M. (2010). Cytotoxicity of the *Bacillus cereus* Nhe enterotoxin requires specific binding order of its three exoprotein components. Infect. Immun..

[14] Zhu K., Didier A., Dietrich R., Heilkenbrinker U., Waltenberger E., Jessberger N., Märtlbauer E., Benz R. (2016). Formation of small transmembrane pores: An intermediate stage on the way to *Bacillus cereus* non-hemolytic enterotoxin (Nhe) full pores in the absence of NheA. Biochem. Biophys. Res. Commun..

[15] Beecher D.J., MacMillan J.D. (1990). A novel bicomponent hemolysin from *Bacillus cereus*. Infect. Immun..

[16] Beecher D.J., MacMillan J.D. (1991). Characterization of the components of hemolysin BL from *Bacillus cereus*. Infect. Immun..

[17] Beecher D.J., Pulido J.S., Barney N.P., Wong A.C. (1995). Extracellular virulence factors in *Bacillus cereus* endophthalmitis: Methods and implication of involvement of hemolysin BL. Infect. Immun..

[18] Beecher D.J., Wong A.C. (1994). Improved purification and characterization of hemolysin BL, a hemolytic dermonecrotic vascular permeability factor from *Bacillus cereus*. Infect. Immun..

[19] Beecher D.J., Wong A.C. (2000). Cooperative, synergistic and antagonistic haemolytic interactions between haemolysin BL, phosphatidylcholine phospholipase C and sphingomyelinase from *Bacillus cereus*. Microbiology.

[20] Jessberger N., Dietrich R., Bock S., Didier A., Märtlbauer E. (2014). *Bacillus cereus* enterotoxins act as major virulence factors and exhibit distinct cytotoxicity to different human cell lines. Toxicon.

[21] Lund T., Granum P.E. (1997). Comparison of biological effect of the two different enterotoxin complexes isolated from three different strains of *Bacillus cereus*. Microbiology.

[22] Sastalla I., Fattah R., Coppage N., Nandy P., Crown D., Pomerantsev A.P., Leppla S.H. (2013). The *Bacillus cereus* Hbl and Nhe tripartite enterotoxin components assemble sequentially on the surface of target cells and are not interchangeable. PLoS ONE.

[23] Mathur A., Feng S., Hayward J.A., Ngo C., Fox D., Atmosukarto I.I., Price J.D., Schauer K., Märtlbauer E., Robertson A.A.B. (2019). A multicomponent toxin from *Bacillus cereus* incites inflammation and shapes host outcome via the NLRP3 inflammasome. Nat. Microbiol..

[24] Beecher D.J., Wong A.C. (1997). Tripartite hemolysin BL from *Bacillus cereus*. Hemolytic analysis of component interactions and a model for its characteristic paradoxical zone phenomenon. J. Biol. Chem..

[25] Jessberger N., Dietrich R., Schwemmer S., Tausch F., Schwenk V., Didier A., Märtlbauer E. (2019). Binding to the Target Cell Surface Is the Crucial Step in Pore Formation of Hemolysin BL from *Bacillus cereus*. Toxins.

[26] Tausch F., Dietrich R., Schauer K., Janowski R., Niessing D., Märtlbauer E., Jessberger N. (2017). Evidence for Complex Formation of the *Bacillus cereus* Haemolysin BL Components in Solution. Toxins.

[27] Lindbäck T., Granum P.E., Alouf J., Ladant D., Popoff M.R. (2015). Bacillus cereus phospholipases, enterotoxins, and other hemolysins. The Comprehensive Sourcebook of Bacterial Protein Toxins.

[28] Margosch D., Maximilian M., Gänzle M.G., Märtlbauer E., Vogel R.F., Ehrmann M.A. (2005). Effect of High Pressure and Heat on Bacterial Toxins. Food Technol. Biotechnol..

[29] Madegowda M., Eswaramoorthy S., Burley S.K., Swaminathan S. (2008). X-ray crystal structure of the B component of Hemolysin BL from *Bacillus cereus*. Proteins.

[30] Benz R., Hancock R.E. (1987). Mechanism of ion transport through the anion-selective channel of the *Pseudomonas aeruginosa* outer membrane. J. Gen. Physiol..

[31] Trias J., Benz R. (1993). Characterization of the channel formed by the mycobacterial porin in lipid bilayer membranes. Demonstration of voltage gating and of negative point charges at the channel mouth. J. Biol. Chem..

[32] Benz R., Popoff M.R. (2018). *Clostridium perfringens* Enterotoxin: The Toxin Forms Highly Cation-Selective Channels in Lipid Bilayers. Toxins.

[33] Benz R., Janko K., Lauger P. (1979). Ionic selectivity of pores formed by the matrix protein (porin) of *Escherichia coli*. Biochim. Biophys. Acta.

[34] Krasilnikov O.V., Kasianowicz J.J., Kellermayer M., Deamer D.W. (2002). Sizing channels with neutral polymers. Structure and Dynamics of Confined Polymers.

[35] Krasilnikov O.V., Da Cruz J.B., Yuldasheva L.N., Varanda W.A., Nogueira R.A. (1998). A novel approach to study the geometry of the water lumen of ion channels: Colicin Ia channels in planar lipid bilayers. J. Membr. Biol..

[36] Krasilnikov O.V., Sabirov R.Z., Ternovsky V.I., Merzliak P.G., Muratkhodjaev J.N. (1992). A simple method for the determination of the pore radius of ion channels in planar lipid bilayer membranes. FEMS Microbiol. Immunol..

[37] Sabirov R.Z., Krasilnikov O.V., Ternovsky V.I., Merzliak P.G. (1993). Relation between ionic channel conductance and conductivity of media containing different nonelectrolytes. A novel method of pore size determination. Gen. Physiol. Biophys..

[38] Bárcena-Uribarri I., Thein M., Maier E., Bonde M., Bergström S., Benz R. (2013). Use of Nonelectrolytes Reveals the Channel Size and Oligomeric Constitution of the *Borrelia burgdorferi* P66 Porin. PLoS ONE.

[39] Holz R., Finkelstein A. (1970). The water and nonelectrolyte permeability induced in thin lipid membranes by the polyene antibiotics nystatin and amphotericin B. J. Gen. Physiol..

[40] Ternovsky V.I., Okada Y., Sabirov R.Z. (2004). Sizing the pore of the volume-sensitive anion channel by differential polymer partitioning. FEBS Lett..

[41] Vodyanoy I., Bezrukov S.M. (1992). Sizing of an ion pore by access resistance measurements. Biophys. J..

[42] Ryan P.A., MacMillan J.D., Zilinskas B.A. (1997). Molecular cloning and characterization of the genes encoding the L1 and L2 components of hemolysin BL from *Bacillus cereus*. J. Bacteriol..

[43] Das B.K., Liang J.J., Chakrabarti B. (1997). Heat-induced conformational change and increased chaperone activity of lens alpha-crystallin. Curr. Eye Res..

[44] Eyles S.J., Gierasch L.M. (2010). Nature’s molecular sponges: Small heat shock proteins grow into their chaperone roles. Proc. Natl. Acad. Sci. USA.

[45] Fu X., Liu C., Liu Y., Feng X., Gu L., Chen X., Chang Z. (2003). Small heat shock protein Hsp16.3 modulates its chaperone activity by adjusting the rate of oligomeric dissociation. Biochem. Biophys. Res. Commun..

[46] Stromer T., Fischer E., Richter K., Haslbeck M., Buchner J. (2004). Analysis of the regulation of the molecular chaperone Hsp26 by temperature-induced dissociation: The N-terminal domail is important for oligomer assembly and the binding of unfolding proteins. J. Biol. Chem..

[47] Ramm F., Dondapati S.K., Thoring L., Zemella A., Wustenhagen D.A., Frentzel H., Stech M., Kubick S. (2020). Mammalian cell-free protein expression promotes the functional characterization of the tripartite non-hemolytic enterotoxin from *Bacillus cereus*. Sci. Rep..

[48] Roderer D., Glockshuber R. (2017). Assembly mechanism of the alpha-pore-forming toxin cytolysin A from *Escherichia coli*. Philos. Trans. R. Soc. B.

[49] Ludwig A., Bauer S., Benz R., Bergmann B., Goebel W. (1999). Analysis of the SlyA-controlled expression, subcellular localization and pore-forming activity of a 34 kDa haemolysin (ClyA) from *Escherichia coli* K-12. Mol. Microbiol..

[50] Oscarsson J., Mizunoe Y., Li L., Lai X.H., Wieslander A., Uhlin B.E. (1999). Molecular analysis of the cytolytic protein ClyA (SheA) from *Escherichia coli*. Mol. Microbiol..

[51] Wallace A.J., Stillman T.J., Atkins A., Jamieson S.J., Bullough P.A., Green J., Artymiuk P.J. (2000). *E. coli* hemolysin E (HlyE, ClyA, SheA): X-ray crystal structure of the toxin and observation of membrane pores by electron microscopy. Cell.

[52] Eifler N., Vetsch M., Gregorini M., Ringler P., Chami M., Philippsen A., Fritz A., Müller S.A., Glockshuber R., Engel A. (2006). Cytotoxin ClyA from *Escherichia coli* assembles to a 13-meric pore independent of its redox-state. EMBO J..

[53] Peng W., de Souza Santos M., Li Y., Tomchick D.R., Orth K. (2019). High-resolution cryo-EM structures of the *E. coli* hemolysin ClyA oligomers. PLoS ONE.

[54] Abdali N., Barth E., Norouzy A., Schulz R., Nau W.M., Kleinekathofer U., Tauch A., Benz R. (2013). *Corynebacterium jeikeium* jk0268 constitutes for the 40 amino acid long PorACj, which forms a homooligomeric and anion-selective cell wall channel. PLoS ONE.

[55] Barth H., Pfeifer G., Hofmann F., Maier E., Benz R., Aktories K. (2001). Low pH-induced formation of ion channels by *Clostridium difficile* toxin B in target cells. J. Biol. Chem..

[56] Clair G., Roussi S., Armengaud J., Duport C. (2010). Expanding the known repertoire of virulence factors produced by *Bacillus cereus* through early secretome profiling in three redox conditions. Mol. Cell. Proteomics.

[57] Gohar M., Faegri K., Perchat S., Ravnum S., Økstad O.A., Gominet M., Kolstø A.B., Lereclus D. (2008). The PlcR virulence regulon of *Bacillus cereus*. PLoS ONE.

[58] Andreeva Z.I., Nesterenko V.F., Fomkina M.G., Ternovsky V.I., Suzina N.E., Bakulina A.Y., Solonin A.S., Sineva E.V. (2007). The properties of *Bacillus cereus* hemolysin II pores depend on environmental conditions. Biochim. Biophys. Acta.

[59] Andreeva Z.I., Nesterenko V.F., Yurkov I.S., Budarina Z.I., Sineva E.V., Solonin A.S. (2006). Purification and cytotoxic properties of *Bacillus cereus* hemolysin II. Protein Expr. Purif..

[60] Ramarao N., Sanchis V. (2013). The pore-forming haemolysins of *Bacillus cereus*: A review. Toxins.

[61] Dietrich R., Fella C., Strich S., Märtlbauer E. (1999). Production and characterization of monoclonal antibodies against the hemolysin BL enterotoxin complex produced by *Bacillus cereus*. Appl. Environ. Microbiol..

[62] Dietrich R., Moravek M., Buerk C., Granum P.E., Märtlbauer E. (2005). Production and characterization of antibodies against each of the three subunits of the *Bacillus cereus* nonhemolytic enterotoxin complex. Appl. Environ. Microbiol..

[63] Jessberger N., Rademacher C., Krey V.M., Dietrich R., Mohr A.K., Böhm M.E., Scherer S., Ehling-Schulz M., Märtlbauer E. (2017). Simulating Intestinal Growth Conditions Enhances Toxin Production of Enteropathogenic *Bacillus cereus*. Front. Microbiol..

[64] Fagerlund A., Lindbäck T., Granum P.E. (2010). *Bacillus cereus* cytotoxins Hbl, Nhe and CytK are secreted via the Sec translocation pathway. BMC Microbiol..

[65] Økstad O.A., Gominet M., Purnelle B., Rose M., Lereclus D., Kolstø A.B. (1999). Sequence analysis of three *Bacillus cereus* loci carrying PIcR-regulated genes encoding degradative enzymes and enterotoxin. Microbiology.

[66] Deutsch E.W., Csordas A., Sun Z., Jarnuczak A., Perez-Riverol Y., Ternent T., Campbell D.S., Bernal-Llinares M., Okuda S., Kawano S. (2017). The ProteomeXchange Consortium in 2017: Supporting the cultural change in proteomics public data deposition. Nucleic Acids Res..

[67] Vizcaíno J.A., Csordas A., del-Toro N., Dianes J.A., Griss J., Lavidas I., Mayer G., Perez-Riverol Y., Reisinger F., Ternent T. (2016). 2016 update of the PRIDE database and related tools. Nucleic Acids Res..

[68] Benz R., Janko K., Boos W., Lauger P. (1978). Formation of large, ion-permeable membrane channels by the matrix protein (porin) of *Escherichia coli*. Biochim. Biophys. Acta.

[69] Janko K., Benz R. (1977). Properties of lipid bilayer membranes made from lipids containing phytanic acid. Biochim. Biophys. Acta.

[70] Scholfield C.R. (1981). Composition of soybean lecithin. J. Am. Oil Chem. Soc..

