# Supplementary material for: Characteristics of the Protein Complexes and Pores Formed by Bacillus cereus Hemolysin BL
Source: Toxins (Basel). 2020 Oct 24;12(11):672. doi: 10.3390/toxins12110672 (PMC7694065; doi:10.3390/toxins12110672)
Supplement: Supplementary file 1 [file toxins-12-00672-s001.pdf]

Supplementary Materials: Characteristics of the Protein Complexes and Pores Formed by *Bacillus cereus* Hemolysin BL

Nadja Jessberger, Richard Dietrich, Kristina Schauer, Stefanie Schwemmer, Erwin Märtlbauer and Roland Benz

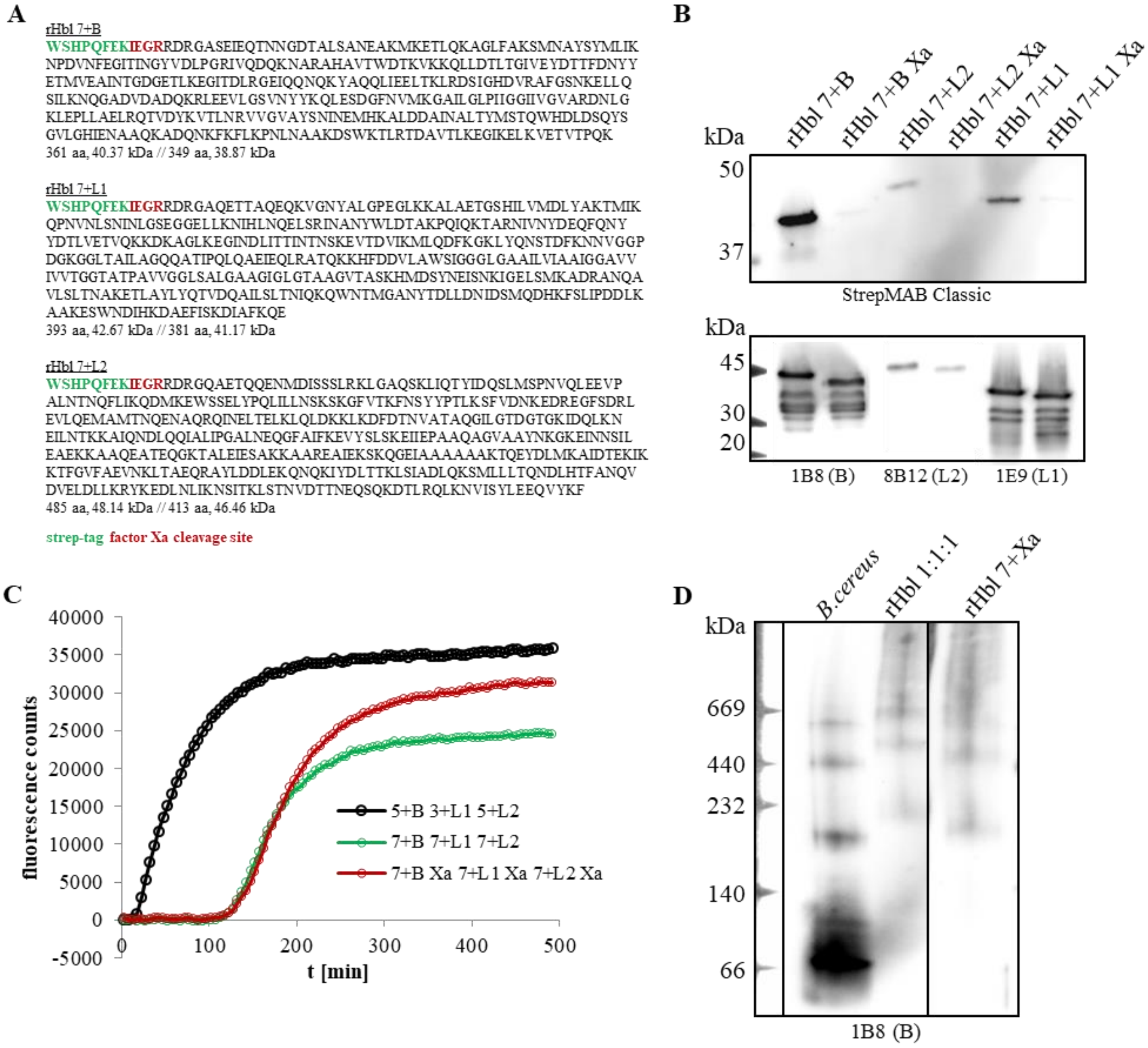

Figure S1. Properties of the rHbl components overexpressed using the pASK-IBA 7+ system**.** (**A**) Sequences of the proteins including strep-tag (green) and factor Xa cleavage site (red). (**B**) Cleavage of the tags shown in Western blots using tag-specific StrepMAB Classic and Hbl-specific mAbs. (**C**) Pore-forming activity of the cleaved proteins. (**D**) Complex formation of the cleaved proteins. rHbl 1:1:1 = concentration ratio of the proteins 5+B, 3+L1 and 5+L2 [26]. 7+Xa = newly generated, cleaved rHbl proteins (this study).

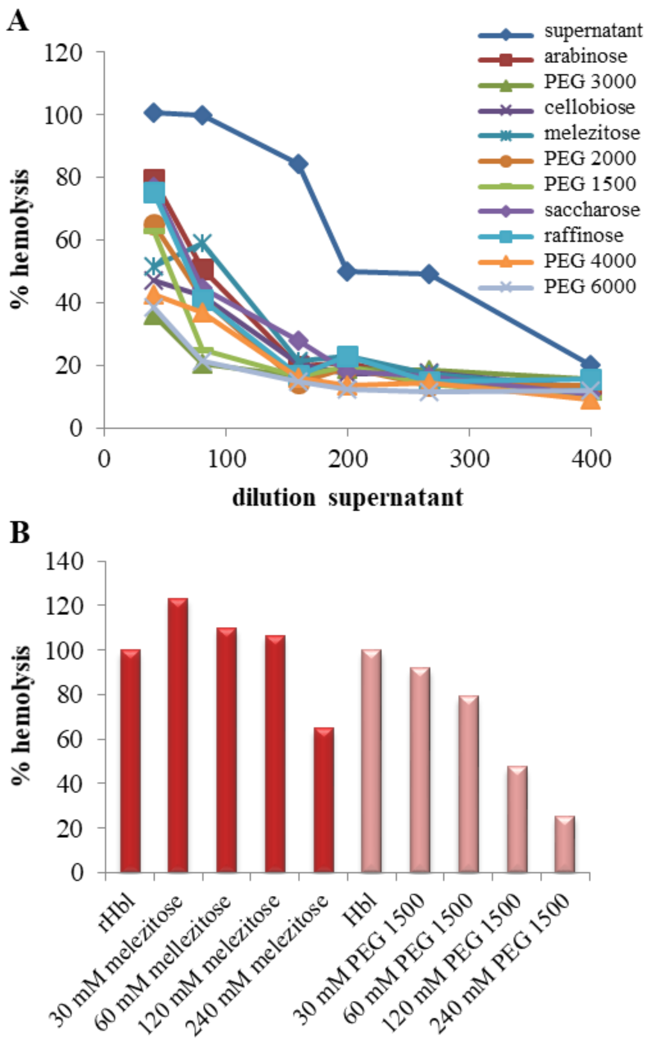

Figure S2. Results of hemolysis assays with defibrinated sheep blood. (**A**) Dilution series of *B. cereus* F837/76 Δ*nheABC* supernatant under addition of 30 mM non-electrolytes. (**B**) Hemolysis of rHbl (1.5 pmol/µl each) under addition of rising concentrations of melezitose and PEG 1500.

Table S1. Results of LC-MS analyses. Excised bands from Coomassie-stained native gels were in-gel digested with trypsin and extracted before NanoLC-MS/MS analyses. Data were compared to UniProt *Bacillus cereus* Reference Proteome database (UP000001417), and the sequences of the Hbl proteins of *B. cereus* strain F837/76. Results are sorted according to their number of significant matches. All results with number of significant matches >5 and number of significant sequences >4 are shown. emPAI: exponentially modified protein abundance index.

**1. *B. cereus* supernatant 700 kDa complex**								
Family	Member	Accession	Score	Mass	Num. of Significant Matches	Num. of Significant Sequences	emPAI	Description
3	1	tr|Q81I63|Q81I63_BACCR	246	109042	19	16	0.93	Microbial collagenase OS=*Bacillus cereus* (strain ATCC 14579 / DSM 31 / JCM 2152 / NBRC 15305 / NCIMB 9373 / NRRL B-3711) OX=226900 GN=BC_0556 PE=4 SV=1
1	1	Hbl_B	271	37838	18	10	3.24	Hbl_B
6	1	tr|Q81EI5|Q81EI5_BACCR	131	43776	17	10	1.88	Putative murein endopeptidase OS=*Bacillus cereus* (strain ATCC 14579 / DSM 31 / JCM 2152 / NBRC 15305 / NCIMB 9373 / NRRL B-3711) OX=226900 GN=BC_1991 PE=4 SV=1
4	1	tr|Q815G3|Q815G3_BACCR	163	47551	9	7	1.03	N-acetylmuramoyl-L-alanine amidase OS=*Bacillus cereus* (strain ATCC 14579 / DSM 31 / JCM 2152 / NBRC 15305 / NCIMB 9373 / NRRL B-3711) OX=226900 GN=BC_5196 PE=4 SV=1
11	1	sp|Q815K8|ENO_BACCR	101	46343	9	7	1.07	Enolase OS=*Bacillus cereus* (strain ATCC 14579 / DSM 31 / JCM 2152 / NBRC 15305 / NCIMB 9373 / NRRL B-3711) OX=226900 GN=eno PE=3 SV=1
14	1	Hbl_L1	92	40669	8	7	1.06	Hbl_L1
8	1	tr|Q81EL6|Q81EL6_BACCR	123	44980	7	7	0.93	Enterotoxin OS=*Bacillus cereus* (strain ATCC 14579 / DSM 31 / JCM 2152 / NBRC 15305 / NCIMB 9373 / NRRL B-3711) OX=226900 GN=BC_1953 PE=4 SV=1
17	1	tr|Q815D0|Q815D0_BACCR	79	60788	7	7	0.63	N-acetylmuramoyl-L-alanine amidase OS=*Bacillus cereus* (strain ATCC 14579 / DSM 31 / JCM 2152 / NBRC 15305 / NCIMB 9373 / NRRL B-3711) OX=226900 GN=BC_5234 PE=4 SV=1
9	1	tr|Q81HW1|Q81HW1_BACCR	118	32367	6	3	0.47	Phospholipase C OS=*Bacillus cereus* (strain ATCC 14579 / DSM 31 / JCM 2152 / NBRC 15305 / NCIMB 9373 / NRRL B-3711) OX=226900 GN=BC_0670 PE=4 SV=1

10	1	tr|Q81HW5|Q81HW5_BACCR	102	87818	6	6	0.33	Immune inhibitor A OS=*Bacillus cereus* (strain ATCC 14579 / DSM 31 / JCM 2152 / NBRC 15305 / NCIMB 9373 / NRRL B-3711) OX=226900 GN=BC_0666 PE=4 SV=1
20	1	Hbl_L2	69	46140	6	6	0.73	Hbl_L2
18	1	tr|Q815P0|Q815P0_BACCR	72	56561	5	5	0.45	Thiol-activated cytolysin OS=*Bacillus cereus* (strain ATCC 14579 / DSM 31 / JCM 2152 / NBRC 15305 / NCIMB 9373 / NRRL B-3711) OX=226900 GN=BC_5101 PE=3 SV=1
**2. *B. cereus* supernatant 600 kDa complex**								
Family	Member	Accession	Score	Mass	Num. of Significant Matches	Num. of Significant Sequences	emPAI	Description
1	1	Hbl_B	326	37838	27	18	9.3	Hbl_B
3	1	tr|Q81I63|Q81I63_BACCR	213	109042	18	11	0.59	Microbial collagenase OS=*Bacillus cereus* (strain ATCC 14579 / DSM 31 / JCM 2152 / NBRC 15305 / NCIMB 9373 / NRRL B-3711) OX=226900 GN=BC_0556 PE=4 SV=1
6	1	Hbl_L2	191	46140	18	15	3.3	Hbl_L2
9	1	tr|Q815G3|Q815G3_BACCR	148	47551	15	14	2.46	N-acetylmuramoyl-L-alanine amidase OS=*Bacillus cereus* (strain ATCC 14579 / DSM 31 / JCM 2152 / NBRC 15305 / NCIMB 9373 / NRRL B-3711) OX=226900 GN=BC_5196 PE=4 SV=1
11	1	tr|Q81BE4|Q81BE4_BACCR	134	43361	15	11	2.2	Surface protein OS=*Bacillus cereus* (strain ATCC 14579 / DSM 31 / JCM 2152 / NBRC 15305 / NCIMB 9373 / NRRL B-3711) OX=226900 GN=BC_3221 PE=4 SV=1
5	1	Hbl_L1	197	40669	14	12	3.26	Hbl_L1
10	1	tr|Q815P0|Q815P0_BACCR	142	56561	12	11	1.44	Thiol-activated cytolysin OS=*Bacillus cereus* (strain ATCC 14579 / DSM 31 / JCM 2152 / NBRC 15305 / NCIMB 9373 / NRRL B-3711) OX=226900 GN=BC_5101 PE=3 SV=1
12	1	tr|Q81HW1|Q81HW1_BACCR	115	32367	11	9	2.65	Phospholipase C OS=*Bacillus cereus* (strain ATCC 14579 / DSM 31 / JCM 2152 / NBRC 15305 / NCIMB 9373 / NRRL B-3711) OX=226900 GN=BC_0670 PE=4 SV=1
8	1	tr|Q815D0|Q815D0_BACCR	153	60788	7	6	0.52	N-acetylmuramoyl-L-alanine amidase OS=*Bacillus cereus* (strain ATCC 14579 / DSM 31 / JCM 2152 / NBRC

								15305 / NCIMB 9373 / NRRL B-3711) OX=226900 GN=BC_5234 PE=4 SV=1
13	1	tr|Q81A83|Q81A83_BACCR	95	36228	7	4	0.59	Antigen OS=*Bacillus cereus* (strain ATCC 14579 / DSM 31 / JCM 2152 / NBRC 15305 / NCIMB 9373 / NRRL B-3711) OX=226900 GN=BC_3699 PE=4 SV=1
17	1	tr|Q81CL9|Q81CL9_BACCR	81	62481	6	5	0.4	Bacillolysin OS=*Bacillus cereus* (strain ATCC 14579 / DSM 31 / JCM 2152 / NBRC 15305 / NCIMB 9373 / NRRL B-3711) OX=226900 GN=BC_2735 PE=4 SV=1
23	1	tr|Q81EI5|Q81EI5_BACCR	51	43776	6	6	0.78	Putative murein endopeptidase OS=*Bacillus cereus* (strain ATCC 14579 / DSM 31 / JCM 2152 / NBRC 15305 / NCIMB 9373 / NRRL B-3711) OX=226900 GN=BC_1991 PE=4 SV=1
1	2	tr|Q7BYC7|Q7BYC7_BACCR	39	52307	6	5	0.5	Hemolysin BL binding component OS=*Bacillus cereus* (strain ATCC 14579 / DSM 31 / JCM 2152 / NBRC 15305 / NCIMB 9373 / NRRL B-3711) OX=226900 GN=BC_3101 PE=4 SV=1
14	1	sp|Q815K8|ENO_BACCR	95	46343	5	5	0.57	Enolase OS=*Bacillus cereus* (strain ATCC 14579 / DSM 31 / JCM 2152 / NBRC 15305 / NCIMB 9373 / NRRL B-3711) OX=226900 GN=eno PE=3 SV=1
16	1	sp|Q814B0|CH60_BACCR	82	57396	5	5	0.44	60 kDa chaperonin OS=*Bacillus cereus* (strain ATCC 14579 / DSM 31 / JCM 2152 / NBRC 15305 / NCIMB 9373 / NRRL B-3711) OX=226900 GN=groL PE=3 SV=1
20	1	tr|Q81EL6|Q81EL6_BACCR	68	44980	5	4	0.6	Enterotoxin OS=*Bacillus cereus* (strain ATCC 14579 / DSM 31 / JCM 2152 / NBRC 15305 / NCIMB 9373 / NRRL B-3711) OX=226900 GN=BC_1953 PE=4 SV=1

3. *B. cereus* supernatant 440 kDa complex

Family	Member	Accession	Score	Mass	Num. of Significant Matches	Num. of Significant Sequences	emPAI	Description
1	1	Hbl_B	303	37838	57	24	21.42	Hbl_B
2	1	tr|Q815P0|Q815P0_BACCR	197	56561	40	24	7.68	Thiol-activated cytolysin OS=*Bacillus cereus* (strain ATCC 14579 / DSM 31 / JCM 2152 / NBRC 15305 / NCIMB 9373 / NRRL B-3711) OX=226900 GN=BC_5101 PE=3 SV=1

4	1	tr|Q81I63|Q81I63_BACCR	179	109042	35	21	1.35	Microbial collagenase OS=*Bacillus cereus* (strain ATCC 14579 / DSM 31 / JCM 2152 / NBRC 15305 / NCIMB 9373 / NRRL B-3711) OX=226900 GN=BC_0556 PE=4 SV=1
3	1	tr|Q81BE4|Q81BE4_BACCR	187	43361	33	22	10.3	Surface protein OS=*Bacillus cereus* (strain ATCC 14579 / DSM 31 / JCM 2152 / NBRC 15305 / NCIMB 9373 / NRRL B-3711) OX=226900 GN=BC_3221 PE=4 SV=1
6	1	tr|Q815G3|Q815G3_BACCR	159	47551	27	18	4.89	N-acetylmuramoyl-L-alanine amidase OS=*Bacillus cereus* (strain ATCC 14579 / DSM 31 / JCM 2152 / NBRC 15305 / NCIMB 9373 / NRRL B-3711) OX=226900 GN=BC_5196 PE=4 SV=1
7	1	tr|Q81HW1|Q81HW1_BACCR	131	32367	23	12	5.13	Phospholipase C OS=*Bacillus cereus* (strain ATCC 14579 / DSM 31 / JCM 2152 / NBRC 15305 / NCIMB 9373 / NRRL B-3711) OX=226900 GN=BC_0670 PE=4 SV=1
10	1	Hbl_L1	113	40669	22	17	4.81	Hbl_L1
9	1	Hbl_L2	118	46140	20	17	3.71	Hbl_L2
11	1	sp|Q815K8|ENO_BACCR	94	46343	13	12	1.97	Enolase OS=*Bacillus cereus* (strain ATCC 14579 / DSM 31 / JCM 2152 / NBRC 15305 / NCIMB 9373 / NRRL B-3711) OX=226900 GN=eno PE=3 SV=1
12	1	tr|Q81HW5|Q81HW5_BACCR	84	87818	13	11	0.7	Immune inhibitor A OS=*Bacillus cereus* (strain ATCC 14579 / DSM 31 / JCM 2152 / NBRC 15305 / NCIMB 9373 / NRRL B-3711) OX=226900 GN=BC_0666 PE=4 SV=1
16	1	tr|Q81CL9|Q81CL9_BACCR	64	62481	12	12	1.25	Bacillolysin OS=*Bacillus cereus* (strain ATCC 14579 / DSM 31 / JCM 2152 / NBRC 15305 / NCIMB 9373 / NRRL B-3711) OX=226900 GN=BC_2735 PE=4 SV=1
13	1	tr|Q81HV2|Q81HV2_BACCR	72	47290	11	10	1.44	Cell wall-binding protein OS=*Bacillus cereus* (strain ATCC 14579 / DSM 31 / JCM 2152 / NBRC 15305 / NCIMB 9373 / NRRL B-3711) OX=226900 GN=BC_0679 PE=4 SV=1
1	2	tr|Q7BYC7|Q7BYC7_BACCR	61	52307	11	7	0.76	Hemolysin BL binding component OS=*Bacillus cereus* (strain ATCC 14579 / DSM 31 / JCM 2152 / NBRC 15305 / NCIMB 9373 / NRRL B-3711) OX=226900 GN=BC_3101 PE=4 SV=1
14	1	tr|Q815D0|Q815D0_BACCR	67	60788	9	8	0.74	N-acetylmuramoyl-L-alanine amidase OS=*Bacillus cereus* (strain ATCC 14579 / DSM 31 / JCM 2152 / NBRC

								15305 / NCIMB 9373 / NRRL B-3711) OX=226900 GN=BC_5234 PE=4 SV=1
15	1	tr|Q81EL6|Q81EL6_BACCR	64	44980	9	7	1.12	Enterotoxin OS=*Bacillus cereus* (strain ATCC 14579 / DSM 31 / JCM 2152 / NBRC 15305 / NCIMB 9373 / NRRL B-3711) OX=226900 GN=BC_1953 PE=4 SV=1
21	1	tr|Q81EI5|Q81EI5_BACCR	45	43776	8	7	1.16	Putative murein endopeptidase OS=*Bacillus cereus* (strain ATCC 14579 / DSM 31 / JCM 2152 / NBRC 15305 / NCIMB 9373 / NRRL B-3711) OX=226900 GN=BC_1991 PE=4 SV=1
17	1	tr|Q81GL0|Q81GL0_BACCR	48	61955	7	7	0.61	Oligopeptide-binding protein oppA OS=*Bacillus cereus* (strain ATCC 14579 / DSM 31 / JCM 2152 / NBRC 15305 / NCIMB 9373 / NRRL B-3711) OX=226900 GN=BC_1185 PE=4 SV=1
24	1	tr|Q81A83|Q81A83_BACCR	42	36228	7	4	0.79	Antigen OS=*Bacillus cereus* (strain ATCC 14579 / DSM 31 / JCM 2152 / NBRC 15305 / NCIMB 9373 / NRRL B-3711) OX=226900 GN=BC_3699 PE=4 SV=1
13	2	tr|Q81HJ4|Q81HJ4_BACCR	50	45574	5	4	0.45	Enterotoxin / cell-wall binding protein OS=*Bacillus cereus* (strain ATCC 14579 / DSM 31 / JCM 2152 / NBRC 15305 / NCIMB 9373 / NRRL B-3711) OX=226900 GN=BC_0813 PE=4 SV=1
**7. *B. cereus* supernatant 120 kDa complex**								
Family	Member	Accession	Score	Mass	Num. of Significant Matches	Num. of Significant Sequences	emPAI	Description
1	1	tr|Q81I63|Q81I63_BACCR	4512	109042	200	45	6.76	Microbial collagenase OS=*Bacillus cereus* (strain ATCC 14579 / DSM 31 / JCM 2152 / NBRC 15305 / NCIMB 9373 / NRRL B-3711) OX=226900 GN=BC_0556 PE=4 SV=1
2	1	Hbl_L2	3731	46140	121	52	215.03	Hbl_L2
3	1	Hbl_L1	2304	40669	85	33	63.04	Hbl_L1
4	1	tr|Q81BE4|Q81BE4_BACCR	1782	43361	78	48	97.44	Surface protein OS=*Bacillus cereus* (strain ATCC 14579 / DSM 31 / JCM 2152 / NBRC 15305 / NCIMB 9373 / NRRL B-3711) OX=226900 GN=BC_3221 PE=4 SV=1
3	2	tr|Q7BYC6|Q7BYC6_BACCR	1638	43804	61	30	17.44	Hemolysin BL lytic component L1 OS=*Bacillus cereus* (strain ATCC 14579 / DSM 31 / JCM 2152 / NBRC 15305

								/ NCIMB 9373 / NRRL B-3711) OX=226900 GN=BC_3103 PE=4 SV=1
5	1	tr|Q81HW0|Q81HW0_BACCR	1572	36900	58	12	2.39	Sphingomyelin phosphodiesterase OS=*Bacillus cereus* (strain ATCC 14579 / DSM 31 / JCM 2152 / NBRC 15305 / NCIMB 9373 / NRRL B-3711) OX=226900 GN=BC_0671 PE=4 SV=1
6	1	tr|Q815P0|Q815P0_BACCR	1486	56561	54	32	11.55	Thiol-activated cytolysin OS=*Bacillus cereus* (strain ATCC 14579 / DSM 31 / JCM 2152 / NBRC 15305 / NCIMB 9373 / NRRL B-3711) OX=226900 GN=BC_5101 PE=3 SV=1
7	1	tr|Q81HW5|Q81HW5_BACCR	1083	87818	45	26	2.64	Immune inhibitor A OS=*Bacillus cereus* (strain ATCC 14579 / DSM 31 / JCM 2152 / NBRC 15305 / NCIMB 9373 / NRRL B-3711) OX=226900 GN=BC_0666 PE=4 SV=1
8	1	Hbl_B	917	37838	42	23	13.53	Hbl_B
10	1	tr|Q81EI5|Q81EI5_BACCR	597	43776	40	15	6.87	Putative murein endopeptidase OS=*Bacillus cereus* (strain ATCC 14579 / DSM 31 / JCM 2152 / NBRC 15305 / NCIMB 9373 / NRRL B-3711) OX=226900 GN=BC_1991 PE=4 SV=1
11	2	tr|Q81HV2|Q81HV2_BACCR	519	47290	29	19	5.23	Cell wall-binding protein OS=*Bacillus cereus* (strain ATCC 14579 / DSM 31 / JCM 2152 / NBRC 15305 / NCIMB 9373 / NRRL B-3711) OX=226900 GN=BC_0679 PE=4 SV=1
9	1	tr|Q81EL6|Q81EL6_BACCR	734	44980	27	13	2.22	Enterotoxin OS=*Bacillus cereus* (strain ATCC 14579 / DSM 31 / JCM 2152 / NBRC 15305 / NCIMB 9373 / NRRL B-3711) OX=226900 GN=BC_1953 PE=4 SV=1
14	1	tr|Q815G3|Q815G3_BACCR	406	47551	22	18	3.5	N-acetylmuramoyl-L-alanine amidase OS=*Bacillus cereus* (strain ATCC 14579 / DSM 31 / JCM 2152 / NBRC 15305 / NCIMB 9373 / NRRL B-3711) OX=226900 GN=BC_5196 PE=4 SV=1
11	1	tr|Q81C32|Q81C32_BACCR	572	55137	20	12	1.6	Enterotoxin / cell-wall binding protein OS=*Bacillus cereus* (strain ATCC 14579 / DSM 31 / JCM 2152 / NBRC 15305 / NCIMB 9373 / NRRL B-3711) OX=226900 GN=BC_2952 PE=4 SV=1

12	1	tr|Q81BN1|Q81BN1_BACCR	566	57895	19	13	1.49	5'-nucleotidase OS=*Bacillus cereus* (strain ATCC 14579 / DSM 31 / JCM 2152 / NBRC 15305 / NCIMB 9373 / NRRL B-3711) OX=226900 GN=BC_3121 PE=3 SV=1
13	1	tr|Q81BS6|Q81BS6_BACCR	453	20592	19	12	9.34	Signal peptidase I OS=*Bacillus cereus* (strain ATCC 14579 / DSM 31 / JCM 2152 / NBRC 15305 / NCIMB 9373 / NRRL B-3711) OX=226900 GN=BC_3070 PE=3 SV=1
17	1	tr|Q81HW1|Q81HW1_BACCR	380	32367	19	10	3	Phospholipase C OS=*Bacillus cereus* (strain ATCC 14579 / DSM 31 / JCM 2152 / NBRC 15305 / NCIMB 9373 / NRRL B-3711) OX=226900 GN=BC_0670 PE=4 SV=1
19	1	tr|Q81A32|Q81A32_BACCR	325	38089	19	9	2.97	1-phosphatidylinositol phosphodiesterase OS=*Bacillus cereus* (strain ATCC 14579 / DSM 31 / JCM 2152 / NBRC 15305 / NCIMB 9373 / NRRL B-3711) OX=226900 GN=BC_3761 PE=4 SV=1
15	1	tr|Q81CL9|Q81CL9_BACCR	403	62481	17	8	0.83	Bacillolysin OS=*Bacillus cereus* (strain ATCC 14579 / DSM 31 / JCM 2152 / NBRC 15305 / NCIMB 9373 / NRRL B-3711) OX=226900 GN=BC_2735 PE=4 SV=1
12	2	tr|Q818Y6|Q818Y6_BACCR	391	57857	16	12	1.19	5'-nucleotidase OS=*Bacillus cereus* (strain ATCC 14579 / DSM 31 / JCM 2152 / NBRC 15305 / NCIMB 9373 / NRRL B-3711) OX=226900 GN=BC_4100 PE=3 SV=1
29	1	tr|Q81AN8|Q81AN8_BACCR	196	45798	14	11	1.47	Hemolysin II OS=*Bacillus cereus* (strain ATCC 14579 / DSM 31 / JCM 2152 / NBRC 15305 / NCIMB 9373 / NRRL B-3711) OX=226900 GN=BC_3523 PE=1 SV=1
16	1	tr|Q815D0|Q815D0_BACCR	388	60788	13	11	0.98	N-acetylmuramoyl-L-alanine amidase OS=*Bacillus cereus* (strain ATCC 14579 / DSM 31 / JCM 2152 / NBRC 15305 / NCIMB 9373 / NRRL B-3711) OX=226900 GN=BC_5234 PE=4 SV=1
18	1	tr|Q819K6|Q819K6_BACCR	341	49409	13	9	0.99	Dihydrolipoyl dehydrogenase OS=*Bacillus cereus* (strain ATCC 14579 / DSM 31 / JCM 2152 / NBRC 15305 / NCIMB 9373 / NRRL B-3711) OX=226900 GN=BC_3970 PE=3 SV=1
10	2	tr|Q81H34|Q81H34_BACCR	272	64559	13	12	1.02	S-layer homology domain / putative murein endopeptidase OS=*Bacillus cereus* (strain ATCC 14579 / DSM 31 / JCM 2152 / NBRC 15305 / NCIMB 9373 / NRRL B-3711) OX=226900 GN=BC_0991 PE=4 SV=1

31	1	tr|Q81HJ1|Q81HJ1_BACCR	184	38925	13	10	2.18	Periplasmic component of efflux system OS=*Bacillus cereus* (strain ATCC 14579 / DSM 31 / JCM 2152 / NBRC 15305 / NCIMB 9373 / NRRL B-3711) OX=226900 GN=BC_0816 PE=3 SV=1
28	1	tr|Q818I3|Q818I3_BACCR	215	79573	12	11	0.69	Penicillin-binding protein OS=*Bacillus cereus* (strain ATCC 14579 / DSM 31 / JCM 2152 / NBRC 15305 / NCIMB 9373 / NRRL B-3711) OX=226900 GN=BC_4270 PE=4 SV=1
20	1	tr|Q81FF4|Q81FF4_BACCR	316	48052	11	7	0.87	Flagellar hook-associated protein 1 OS=*Bacillus cereus* (strain ATCC 14579 / DSM 31 / JCM 2152 / NBRC 15305 / NCIMB 9373 / NRRL B-3711) OX=226900 GN=BC_1636 PE=4 SV=1
23	1	sp|Q814C5|EFG_BACCR	257	76276	11	10	0.64	Elongation factor G OS=*Bacillus cereus* (strain ATCC 14579 / DSM 31 / JCM 2152 / NBRC 15305 / NCIMB 9373 / NRRL B-3711) OX=226900 GN=fusA PE=3 SV=1
24	1	tr|Q81IA3|Q81IA3_BACCR	254	84627	10	7	0.5	Formate acetyltransferase OS=*Bacillus cereus* (strain ATCC 14579 / DSM 31 / JCM 2152 / NBRC 15305 / NCIMB 9373 / NRRL B-3711) OX=226900 GN=BC_0491 PE=4 SV=1
33	1	tr|Q81EK1|Q81EK1_BACCR	176	37744	10	9	1.45	Transcriptional regulator, LytR family OS=*Bacillus cereus* (strain ATCC 14579 / DSM 31 / JCM 2152 / NBRC 15305 / NCIMB 9373 / NRRL B-3711) OX=226900 GN=BC_1969 PE=4 SV=1
34	1	tr|Q817F4|Q817F4_BACCR	171	62229	10	7	0.53	Pyruvate kinase OS=*Bacillus cereus* (strain ATCC 14579 / DSM 31 / JCM 2152 / NBRC 15305 / NCIMB 9373 / NRRL B-3711) OX=226900 GN=BC_4599 PE=3 SV=1
44	1	tr|Q81AR2|Q81AR2_BACCR	126	35823	10	8	1.56	Glycerophosphoryl diester phosphodiesterase OS=*Bacillus cereus* (strain ATCC 14579 / DSM 31 / JCM 2152 / NBRC 15305 / NCIMB 9373 / NRRL B-3711) OX=226900 GN=BC_3495 PE=4 SV=1
22	1	tr|Q81AG8|Q81AG8_BACCR	260	43880	9	8	0.99	Protease HhoA OS=*Bacillus cereus* (strain ATCC 14579 / DSM 31 / JCM 2152 / NBRC 15305 / NCIMB 9373 / NRRL B-3711) OX=226900 GN=BC_3600 PE=4 SV=1

26	1	tr|Q81CG6|Q81CG6_BACCR	228	49700	9	7	0.7	Chitin binding protein OS=*Bacillus cereus* (strain ATCC 14579 / DSM 31 / JCM 2152 / NBRC 15305 / NCIMB 9373 / NRRL B-3711) OX=226900 GN=BC_2798 PE=4 SV=1
30	1	tr|Q81HU4|Q81HU4_BACCR	195	56150	9	8	0.71	IG hypothetical 22578 OS=*Bacillus cereus* (strain ATCC 14579 / DSM 31 / JCM 2152 / NBRC 15305 / NCIMB 9373 / NRRL B-3711) OX=226900 GN=BC_0687 PE=4 SV=1
11	3	tr|Q81HJ4|Q81HJ4_BACCR	151	45574	9	8	0.93	Enterotoxin / cell-wall binding protein OS=*Bacillus cereus* (strain ATCC 14579 / DSM 31 / JCM 2152 / NBRC 15305 / NCIMB 9373 / NRRL B-3711) OX=226900 GN=BC_0813 PE=4 SV=1
52	1	tr|Q7BYC7|Q7BYC7_BACCR	108	52307	9	9	0.91	Hemolysin BL binding component OS=*Bacillus cereus* (strain ATCC 14579 / DSM 31 / JCM 2152 / NBRC 15305 / NCIMB 9373 / NRRL B-3711) OX=226900 GN=BC_3101 PE=4 SV=1
1	2	tr|Q81AN3|Q81AN3_BACCR	102	109016	8	4	0.15	Microbial collagenase OS=*Bacillus cereus* (strain ATCC 14579 / DSM 31 / JCM 2152 / NBRC 15305 / NCIMB 9373 / NRRL B-3711) OX=226900 GN=BC_3529 PE=4 SV=1
37	1	tr|Q81B73|Q81B73_BACCR	160	29277	7	5	0.89	6-aminohexanoate-dimer hydrolase OS=*Bacillus cereus* (strain ATCC 14579 / DSM 31 / JCM 2152 / NBRC 15305 / NCIMB 9373 / NRRL B-3711) OX=226900 GN=BC_3315 PE=4 SV=1
50	1	tr|Q81C49|Q81C49_BACCR	111	74607	7	7	0.43	Phosphoglycerol transferase OS=*Bacillus cereus* (strain ATCC 14579 / DSM 31 / JCM 2152 / NBRC 15305 / NCIMB 9373 / NRRL B-3711) OX=226900 GN=BC_2932 PE=3 SV=1
36	1	tr|Q813P2|Q813P2_BACCR	163	16669	6	4	2.03	Non-specific DNA-binding protein Dps / Iron-binding ferritin-like antioxidant protein / Ferroxidase OS=*Bacillus cereus* (strain ATCC 14579 / DSM 31 / JCM 2152 / NBRC 15305 / NCIMB 9373 / NRRL B-3711) OX=226900 GN=BC_2011 PE=3 SV=1
38	1	tr|Q81AM9|Q81AM9_BACCR	154	31429	6	4	0.81	Vancomycin B-type resistance protein vanW OS=*Bacillus cereus* (strain ATCC 14579 / DSM 31 / JCM 2152 / NBRC 15305 / NCIMB 9373 / NRRL B-3711) OX=226900 GN=BC_3533 PE=4 SV=1

39	1	tr|Q81FY1|Q81FY1_BACCR	144	46755	6	4	0.38	Cell wall endopeptidase, family M23/M37 OS=*Bacillus cereus* (strain ATCC 14579 / DSM 31 / JCM 2152 / NBRC 15305 / NCIMB 9373 / NRRL B-3711) OX=226900 GN=BC_1431 PE=4 SV=1
40	1	tr|Q81DA0|Q81DA0_BACCR	144	33964	6	4	0.55	Beta-lactamase OS=*Bacillus cereus* (strain ATCC 14579 / DSM 31 / JCM 2152 / NBRC 15305 / NCIMB 9373 / NRRL B-3711) OX=226900 GN=BC_2473 PE=3 SV=1
41	1	tr|Q81FE1|Q81FE1_BACCR	143	46203	6	4	0.5	Flagellar hook protein FlgE OS=*Bacillus cereus* (strain ATCC 14579 / DSM 31 / JCM 2152 / NBRC 15305 / NCIMB 9373 / NRRL B-3711) OX=226900 GN=BC_1651 PE=3 SV=1
61	1	tr|Q815U2|Q815U2_BACCR	69	60557	6	5	0.36	Bacillolysin OS=*Bacillus cereus* (strain ATCC 14579 / DSM 31 / JCM 2152 / NBRC 15305 / NCIMB 9373 / NRRL B-3711) OX=226900 GN=BC_5036 PE=4 SV=1

9. *B. cereus* supernatant 70 kDa complex

Family	Member	Accession	Score	Mass	Num. of Significant Matches	Num. of Significant Sequences	emPAI	Description
1	1	Hbl_B	6150	37838	186	51	514.23	Hbl_B
2	1	tr|Q81I63|Q81I63_BACCR	4551	109042	122	42	6.24	Microbial collagenase OS=*Bacillus cereus* (strain ATCC 14579 / DSM 31 / JCM 2152 / NBRC 15305 / NCIMB 9373 / NRRL B-3711) OX=226900 GN=BC_0556 PE=4 SV=1
3	1	tr|Q815P0|Q815P0_BACCR	3233	56561	86	41	26.89	Thiol-activated cytolysin OS=*Bacillus cereus* (strain ATCC 14579 / DSM 31 / JCM 2152 / NBRC 15305 / NCIMB 9373 / NRRL B-3711) OX=226900 GN=BC_5101 PE=3 SV=1
5	1	tr|Q81BE4|Q81BE4_BACCR	2164	43361	83	50	126.64	Surface protein OS=*Bacillus cereus* (strain ATCC 14579 / DSM 31 / JCM 2152 / NBRC 15305 / NCIMB 9373 / NRRL B-3711) OX=226900 GN=BC_3221 PE=4 SV=1
8	1	tr|Q81EI5|Q81EI5_BACCR	1780	43776	70	18	12.17	Putative murein endopeptidase OS=*Bacillus cereus* (strain ATCC 14579 / DSM 31 / JCM 2152 / NBRC 15305 / NCIMB 9373 / NRRL B-3711) OX=226900 GN=BC_1991 PE=4 SV=1
4	1	Hbl_L2	2521	46140	67	39	41.37	Hbl_L2

7	1	tr|Q815D2|Q815D2_BACCR	1801	72036	51	26	4.94	Phosphoglycerol transferase OS=*Bacillus cereus* (strain ATCC 14579 / DSM 31 / JCM 2152 / NBRC 15305 / NCIMB 9373 / NRRL B-3711) OX=226900 GN=BC_5232 PE=3 SV=1
6	1	Hbl_L1	2078	40669	47	27	16.56	Hbl_L1
12	1	tr|Q81HW5|Q81HW5_BACCR	1275	87818	42	27	2.64	Immune inhibitor A OS=*Bacillus cereus* (strain ATCC 14579 / DSM 31 / JCM 2152 / NBRC 15305 / NCIMB 9373 / NRRL B-3711) OX=226900 GN=BC_0666 PE=4 SV=1
10	1	tr|Q81EL6|Q81EL6_BACCR	1592	44980	39	16	3.51	Enterotoxin OS=*Bacillus cereus* (strain ATCC 14579 / DSM 31 / JCM 2152 / NBRC 15305 / NCIMB 9373 / NRRL B-3711) OX=226900 GN=BC_1953 PE=4 SV=1
11	1	tr|Q81CL9|Q81CL9_BACCR	1481	62481	39	14	1.96	Bacillolysin OS=*Bacillus cereus* (strain ATCC 14579 / DSM 31 / JCM 2152 / NBRC 15305 / NCIMB 9373 / NRRL B-3711) OX=226900 GN=BC_2735 PE=4 SV=1
9	1	tr|Q81C49|Q81C49_BACCR	1710	74607	38	20	2.55	Phosphoglycerol transferase OS=*Bacillus cereus* (strain ATCC 14579 / DSM 31 / JCM 2152 / NBRC 15305 / NCIMB 9373 / NRRL B-3711) OX=226900 GN=BC_2932 PE=3 SV=1
16	1	sp|Q814C5|EFG_BACCR	969	76276	34	21	2.45	Elongation factor G OS=*Bacillus cereus* (strain ATCC 14579 / DSM 31 / JCM 2152 / NBRC 15305 / NCIMB 9373 / NRRL B-3711) OX=226900 GN=fusA PE=3 SV=1
18	1	tr|Q81HW1|Q81HW1_BACCR	745	32367	33	15	7	Phospholipase C OS=*Bacillus cereus* (strain ATCC 14579 / DSM 31 / JCM 2152 / NBRC 15305 / NCIMB 9373 / NRRL B-3711) OX=226900 GN=BC_0670 PE=4 SV=1
14	1	tr|Q81FF4|Q81FF4_BACCR	1025	48052	32	15	3.79	Flagellar hook-associated protein 1 OS=*Bacillus cereus* (strain ATCC 14579 / DSM 31 / JCM 2152 / NBRC 15305 / NCIMB 9373 / NRRL B-3711) OX=226900 GN=BC_1636 PE=4 SV=1
8	2	tr|Q81H34|Q81H34_BACCR	1007	64559	30	20	2.22	S-layer homology domain / putative murein endopeptidase OS=*Bacillus cereus* (strain ATCC 14579 / DSM 31 / JCM 2152 / NBRC 15305 / NCIMB 9373 / NRRL B-3711) OX=226900 GN=BC_0991 PE=4 SV=1
15	1	tr|Q815U2|Q815U2_BACCR	998	60557	29	20	2.69	Bacillolysin OS=*Bacillus cereus* (strain ATCC 14579 / DSM 31 / JCM 2152 / NBRC 15305 / NCIMB 9373 / NRRL B-3711) OX=226900 GN=BC_5036 PE=4 SV=1

13	1	tr|Q81C32|Q81C32_BACCR	1074	55137	28	13	2.19	Enterotoxin / cell-wall binding protein OS=*Bacillus cereus* (strain ATCC 14579 / DSM 31 / JCM 2152 / NBRC 15305 / NCIMB 9373 / NRRL B-3711) OX=226900 GN=BC_2952 PE=4 SV=1
20	1	tr|Q81AN8|Q81AN8_BACCR	719	45798	23	18	3.75	Hemolysin II OS=*Bacillus cereus* (strain ATCC 14579 / DSM 31 / JCM 2152 / NBRC 15305 / NCIMB 9373 / NRRL B-3711) OX=226900 GN=BC_3523 PE=1 SV=1
19	1	tr|Q81BN1|Q81BN1_BACCR	743	57895	21	15	1.65	5'-nucleotidase OS=*Bacillus cereus* (strain ATCC 14579 / DSM 31 / JCM 2152 / NBRC 15305 / NCIMB 9373 / NRRL B-3711) OX=226900 GN=BC_3121 PE=3 SV=1
17	1	tr|Q81AG8|Q81AG8_BACCR	770	43880	18	12	1.8	Protease HhoA OS=*Bacillus cereus* (strain ATCC 14579 / DSM 31 / JCM 2152 / NBRC 15305 / NCIMB 9373 / NRRL B-3711) OX=226900 GN=BC_3600 PE=4 SV=1
22	1	tr|Q81FZ5|Q81FZ5_BACCR	649	72235	18	12	0.87	Phosphoglycerol transferase OS=*Bacillus cereus* (strain ATCC 14579 / DSM 31 / JCM 2152 / NBRC 15305 / NCIMB 9373 / NRRL B-3711) OX=226900 GN=BC_1417 PE=3 SV=1
23	1	tr|Q814T5|Q814T5_BACCR	575	30653	18	10	3.33	Fructose-bisphosphate aldolase OS=*Bacillus cereus* (strain ATCC 14579 / DSM 31 / JCM 2152 / NBRC 15305 / NCIMB 9373 / NRRL B-3711) OX=226900 GN=BC_5335 PE=4 SV=1
19	2	tr|Q818Y6|Q818Y6_BACCR	519	57857	16	10	1.05	5'-nucleotidase OS=*Bacillus cereus* (strain ATCC 14579 / DSM 31 / JCM 2152 / NBRC 15305 / NCIMB 9373 / NRRL B-3711) OX=226900 GN=BC_4100 PE=3 SV=1
50	1	tr|Q815G3|Q815G3_BACCR	223	47551	16	12	1.8	N-acetylmuramoyl-L-alanine amidase OS=*Bacillus cereus* (strain ATCC 14579 / DSM 31 / JCM 2152 / NBRC 15305 / NCIMB 9373 / NRRL B-3711) OX=226900 GN=BC_5196 PE=4 SV=1
21	1	sp|Q812X3|EFTS_BACCR	651	32459	15	12	3	Elongation factor Ts OS=*Bacillus cereus* (strain ATCC 14579 / DSM 31 / JCM 2152 / NBRC 15305 / NCIMB 9373 / NRRL B-3711) OX=226900 GN=tsf PE=3 SV=1
24	1	tr|Q815D0|Q815D0_BACCR	488	60788	15	11	1.1	N-acetylmuramoyl-L-alanine amidase OS=*Bacillus cereus* (strain ATCC 14579 / DSM 31 / JCM 2152 / NBRC 15305 / NCIMB 9373 / NRRL B-3711) OX=226900 GN=BC_5234 PE=4 SV=1

33	1	sp|Q815K8|ENO_BACCR	354	46343	15	12	1.65	Enolase OS=*Bacillus cereus* (strain ATCC 14579 / DSM 31 / JCM 2152 / NBRC 15305 / NCIMB 9373 / NRRL B-3711) OX=226900 GN=eno PE=3 SV=1
1	2	tr|Q7BYC7|Q7BYC7_BACCR	264	52307	14	13	1.55	Hemolysin BL binding component OS=*Bacillus cereus* (strain ATCC 14579 / DSM 31 / JCM 2152 / NBRC 15305 / NCIMB 9373 / NRRL B-3711) OX=226900 GN=BC_3101 PE=4 SV=1
30	1	tr|Q81A32|Q81A32_BACCR	424	38089	13	7	1.96	1-phosphatidylinositol phosphodiesterase OS=*Bacillus cereus* (strain ATCC 14579 / DSM 31 / JCM 2152 / NBRC 15305 / NCIMB 9373 / NRRL B-3711) OX=226900 GN=BC_3761 PE=4 SV=1
32	1	tr|Q81HU4|Q81HU4_BACCR	354	56150	13	12	1.39	IG hypothetical 22578 OS=*Bacillus cereus* (strain ATCC 14579 / DSM 31 / JCM 2152 / NBRC 15305 / NCIMB 9373 / NRRL B-3711) OX=226900 GN=BC_0687 PE=4 SV=1
36	1	tr|Q81EK1|Q81EK1_BACCR	329	37744	13	10	1.7	Transcriptional regulator, LytR family OS=*Bacillus cereus* (strain ATCC 14579 / DSM 31 / JCM 2152 / NBRC 15305 / NCIMB 9373 / NRRL B-3711) OX=226900 GN=BC_1969 PE=4 SV=1
25	1	tr|Q819K6|Q819K6_BACCR	457	49409	12	9	0.99	Dihydrolipoyl dehydrogenase OS=*Bacillus cereus* (strain ATCC 14579 / DSM 31 / JCM 2152 / NBRC 15305 / NCIMB 9373 / NRRL B-3711) OX=226900 GN=BC_3970 PE=3 SV=1
29	1	tr|Q815C5|Q815C5_BACCR	427	31221	12	6	1.61	Enterotoxin / cell-wall binding protein OS=*Bacillus cereus* (strain ATCC 14579 / DSM 31 / JCM 2152 / NBRC 15305 / NCIMB 9373 / NRRL B-3711) OX=226900 GN=BC_5239 PE=4 SV=1
42	1	tr|Q81HJ1|Q81HJ1_BACCR	281	38925	12	10	1.62	Periplasmic component of efflux system OS=*Bacillus cereus* (strain ATCC 14579 / DSM 31 / JCM 2152 / NBRC 15305 / NCIMB 9373 / NRRL B-3711) OX=226900 GN=BC_0816 PE=3 SV=1
45	1	tr|Q81BS6|Q81BS6_BACCR	250	20592	12	10	5.03	Signal peptidase I OS=*Bacillus cereus* (strain ATCC 14579 / DSM 31 / JCM 2152 / NBRC 15305 / NCIMB 9373 / NRRL B-3711) OX=226900 GN=BC_3070 PE=3 SV=1

46	1	tr|Q81HV2|Q81HV2_BACCR	248	47290	12	8	1.05	Cell wall-binding protein OS=*Bacillus cereus* (strain ATCC 14579 / DSM 31 / JCM 2152 / NBRC 15305 / NCIMB 9373 / NRRL B-3711) OX=226900 GN=BC_0679 PE=4 SV=1
26	1	sp|Q818E9|DNAK_BACCR	445	65726	11	9	0.68	Chaperone protein DnaK OS=*Bacillus cereus* (strain ATCC 14579 / DSM 31 / JCM 2152 / NBRC 15305 / NCIMB 9373 / NRRL B-3711) OX=226900 GN=dnaK PE=3 SV=1
44	1	tr|Q818I3|Q818I3_BACCR	259	79573	11	8	0.61	Penicillin-binding protein OS=*Bacillus cereus* (strain ATCC 14579 / DSM 31 / JCM 2152 / NBRC 15305 / NCIMB 9373 / NRRL B-3711) OX=226900 GN=BC_4270 PE=4 SV=1
27	1	tr|Q81HW0|Q81HW0_BACCR	431	36900	10	6	0.84	Sphingomyelin phosphodiesterase OS=*Bacillus cereus* (strain ATCC 14579 / DSM 31 / JCM 2152 / NBRC 15305 / NCIMB 9373 / NRRL B-3711) OX=226900 GN=BC_0671 PE=4 SV=1
39	1	tr|Q81AM9|Q81AM9_BACCR	304	31429	10	8	1.92	Vancomycin B-type resistance protein vanW OS=*Bacillus cereus* (strain ATCC 14579 / DSM 31 / JCM 2152 / NBRC 15305 / NCIMB 9373 / NRRL B-3711) OX=226900 GN=BC_3533 PE=4 SV=1
40	1	tr|Q815H1|Q815H1_BACCR	292	52395	10	9	0.91	Tail-specific protease OS=*Bacillus cereus* (strain ATCC 14579 / DSM 31 / JCM 2152 / NBRC 15305 / NCIMB 9373 / NRRL B-3711) OX=226900 GN=BC_5184 PE=3 SV=1
31	1	tr|Q81C52|Q81C52_BACCR	414	30805	9	8	1.98	Peptidoglycan N-acetylglucosamine deacetylase OS=*Bacillus cereus* (strain ATCC 14579 / DSM 31 / JCM 2152 / NBRC 15305 / NCIMB 9373 / NRRL B-3711) OX=226900 GN=BC_2929 PE=4 SV=1
43	1	tr|Q81A13|Q81A13_BACCR	260	33571	9	7	1.19	Transcriptional regulator OS=*Bacillus cereus* (strain ATCC 14579 / DSM 31 / JCM 2152 / NBRC 15305 / NCIMB 9373 / NRRL B-3711) OX=226900 GN=BC_3782 PE=4 SV=1
49	1	sp|Q819X9|RS2_BACCR	227	26546	9	7	2.08	30S ribosomal protein S2 OS=*Bacillus cereus* (strain ATCC 14579 / DSM 31 / JCM 2152 / NBRC 15305 / NCIMB 9373 / NRRL B-3711) OX=226900 GN=rpsB PE=3 SV=1

58	1	tr|Q81FD2|Q81FD2_BACCR	176	28055	9	7	1.9	Soluble lytic murein transglycosylase OS=*Bacillus cereus* (strain ATCC 14579 / DSM 31 / JCM 2152 / NBRC 15305 / NCIMB 9373 / NRRL B-3711) OX=226900 GN=BC_1660 PE=4 SV=1
34	1	tr|Q81IA3|Q81IA3_BACCR	352	84627	8	6	0.37	Formate acetyltransferase OS=*Bacillus cereus* (strain ATCC 14579 / DSM 31 / JCM 2152 / NBRC 15305 / NCIMB 9373 / NRRL B-3711) OX=226900 GN=BC_0491 PE=4 SV=1
38	1	tr|Q81FL5|Q81FL5_BACCR	314	58924	8	6	0.47	Metal-dependent carboxypeptidase OS=*Bacillus cereus* (strain ATCC 14579 / DSM 31 / JCM 2152 / NBRC 15305 / NCIMB 9373 / NRRL B-3711) OX=226900 GN=BC_1566 PE=3 SV=1
29	2	tr|Q81HJ4|Q81HJ4_BACCR	288	45574	8	8	0.93	Enterotoxin / cell-wall binding protein OS=*Bacillus cereus* (strain ATCC 14579 / DSM 31 / JCM 2152 / NBRC 15305 / NCIMB 9373 / NRRL B-3711) OX=226900 GN=BC_0813 PE=4 SV=1
51	1	tr|Q817M4|Q817M4_BACCR	223	29372	8	3	0.66	D-alanyl-D-alanine carboxypeptidase OS=*Bacillus cereus* (strain ATCC 14579 / DSM 31 / JCM 2152 / NBRC 15305 / NCIMB 9373 / NRRL B-3711) OX=226900 GN=BC_4514 PE=4 SV=1
54	1	tr|Q81HR4|Q81HR4_BACCR	189	42120	8	6	0.71	Cell wall endopeptidase, family M23/M37 OS=*Bacillus cereus* (strain ATCC 14579 / DSM 31 / JCM 2152 / NBRC 15305 / NCIMB 9373 / NRRL B-3711) OX=226900 GN=BC_0740 PE=4 SV=1
35	1	tr|Q81FY1|Q81FY1_BACCR	333	46755	7	5	0.5	Cell wall endopeptidase, family M23/M37 OS=*Bacillus cereus* (strain ATCC 14579 / DSM 31 / JCM 2152 / NBRC 15305 / NCIMB 9373 / NRRL B-3711) OX=226900 GN=BC_1431 PE=4 SV=1
47	1	sp|Q816G0|G6PI_BACCR	231	50280	7	7	0.69	Glucose-6-phosphate isomerase OS=*Bacillus cereus* (strain ATCC 14579 / DSM 31 / JCM 2152 / NBRC 15305 / NCIMB 9373 / NRRL B-3711) OX=226900 GN=pgi PE=3 SV=1
52	1	tr|Q813P2|Q813P2_BACCR	212	16669	7	3	2.03	Non-specific DNA-binding protein Dps / Iron-binding ferritin-like antioxidant protein / Ferroxidase OS=*Bacillus cereus* (strain ATCC 14579 / DSM 31 / JCM

								2152 / NBRC 15305 / NCIMB 9373 / NRRL B-3711) OX=226900 GN=BC_2011 PE=3 SV=1
55	1	sp|Q819J5|DAPH_BACCR	180	25689	7	5	1.39	2,3,4,5-tetrahydropyridine-2,6-dicarboxylate N-acetyltransferase OS=*Bacillus cereus* (strain ATCC 14579 / DSM 31 / JCM 2152 / NBRC 15305 / NCIMB 9373 / NRRL B-3711) OX=226900 GN=dapH PE=3 SV=1
56	1	tr|Q81DC9|Q81DC9_BACCR	177	54545	7	7	0.62	Aminoacyl-histidine dipeptidase OS=*Bacillus cereus* (strain ATCC 14579 / DSM 31 / JCM 2152 / NBRC 15305 / NCIMB 9373 / NRRL B-3711) OX=226900 GN=BC_2439 PE=4 SV=1
62	1	tr|Q81HK5|Q81HK5_BACCR	163	45535	7	6	0.64	Transcriptional regulator, LytR family OS=*Bacillus cereus* (strain ATCC 14579 / DSM 31 / JCM 2152 / NBRC 15305 / NCIMB 9373 / NRRL B-3711) OX=226900 GN=BC_0801 PE=4 SV=1
75	1	tr|Q81FF2|Q81FF2_BACCR	120	50659	7	2	0.25	Flagellar hook-associated protein 2 OS=*Bacillus cereus* (strain ATCC 14579 / DSM 31 / JCM 2152 / NBRC 15305 / NCIMB 9373 / NRRL B-3711) OX=226900 GN=BC_1638 PE=3 SV=1
41	1	tr|Q815K6|Q815K6_BACCR	284	35176	6	4	0.7	Glyceraldehyde-3-phosphate dehydrogenase OS=*Bacillus cereus* (strain ATCC 14579 / DSM 31 / JCM 2152 / NBRC 15305 / NCIMB 9373 / NRRL B-3711) OX=226900 GN=BC_5140 PE=3 SV=1
53	1	tr|Q81E10|Q81E10_BACCR	194	22165	6	4	0.95	Uncharacterized protein OS=*Bacillus cereus* (strain ATCC 14579 / DSM 31 / JCM 2152 / NBRC 15305 / NCIMB 9373 / NRRL B-3711) OX=226900 GN=BC_2186 PE=4 SV=1
57	1	sp|Q81J39|RL2_BACCR	177	30260	6	6	1.1	50S ribosomal protein L2 OS=*Bacillus cereus* (strain ATCC 14579 / DSM 31 / JCM 2152 / NBRC 15305 / NCIMB 9373 / NRRL B-3711) OX=226900 GN=rplB PE=3 SV=1
66	1	sp|Q81GI3|FABI_BACCR	146	27723	6	5	0.96	Enoyl-[acyl-carrier-protein] reductase [NADH] FabI OS=*Bacillus cereus* (strain ATCC 14579 / DSM 31 / JCM 2152 / NBRC 15305 / NCIMB 9373 / NRRL B-3711) OX=226900 GN=fabI PE=1 SV=1

65	1	tr|Q81CG6|Q81CG6_BACCR	146	49700	6	5	0.46	Chitin binding protein OS=*Bacillus cereus* (strain ATCC 14579 / DSM 31 / JCM 2152 / NBRC 15305 / NCIMB 9373 / NRRL B-3711) OX=226900 GN=BC_2798 PE=4 SV=1
2	2	tr|Q81AN3|Q81AN3_BACCR	120	109016	6	4	0.15	Microbial collagenase OS=*Bacillus cereus* (strain ATCC 14579 / DSM 31 / JCM 2152 / NBRC 15305 / NCIMB 9373 / NRRL B-3711) OX=226900 GN=BC_3529 PE=4 SV=1
76	1	tr|Q81IX8|Q81IX8_BACCR	119	61463	6	5	0.36	Oligopeptide-binding protein oppA OS=*Bacillus cereus* (strain ATCC 14579 / DSM 31 / JCM 2152 / NBRC 15305 / NCIMB 9373 / NRRL B-3711) OX=226900 GN=BC_0216 PE=4 SV=1
79	1	sp|Q816T0|SYL_BACCR	113	91296	6	6	0.28	Leucine--tRNA ligase OS=*Bacillus cereus* (strain ATCC 14579 / DSM 31 / JCM 2152 / NBRC 15305 / NCIMB 9373 / NRRL B-3711) OX=226900 GN=leuS PE=3 SV=1
61	1	tr|Q81A83|Q81A83_BACCR	168	36228	5	5	0.68	Antigen OS=*Bacillus cereus* (strain ATCC 14579 / DSM 31 / JCM 2152 / NBRC 15305 / NCIMB 9373 / NRRL B-3711) OX=226900 GN=BC_3699 PE=4 SV=1
63	1	sp|Q819H7|METE_BACCR	157	87131	5	5	0.24	5-methyltetrahydropteroyltriglutamate--homocysteine methyltransferase OS=*Bacillus cereus* (strain ATCC 14579 / DSM 31 / JCM 2152 / NBRC 15305 / NCIMB 9373 / NRRL B-3711) OX=226900 GN=metE PE=3 SV=1
73	1	sp|P60180|TPIS_BACCR	125	26452	5	4	1.03	Triosephosphate isomerase OS=*Bacillus cereus* (strain ATCC 14579 / DSM 31 / JCM 2152 / NBRC 15305 / NCIMB 9373 / NRRL B-3711) OX=226900 GN=tpiA PE=5 SV=1
74	1	sp|Q81J61|SYE_BACCR	120	55669	5	4	0.31	Glutamate--tRNA ligase OS=*Bacillus cereus* (strain ATCC 14579 / DSM 31 / JCM 2152 / NBRC 15305 / NCIMB 9373 / NRRL B-3711) OX=226900 GN=gltX PE=3 SV=2
**10. rHbl 440 kDa complex**								
Family	Member	Accession	Score	Mass	Num. of Significant Matches	Num. of Significant Sequences	emPAI	Description
3	1	Hbl_B	2288	37838	130	33	83.93	Hbl_B
1	1	Hbl_L2	3117	46140	127	52	341.64	Hbl_L2
2	1	Hbl_L1	2523	40669	119	35	104.54	Hbl_L1

2	2	tr|Q7BYC6|Q7BYC6_BACCR	2196	43804	93	35	37.4	Hemolysin BL lytic component L1 OS=*Bacillus cereus* (strain ATCC 14579 / DSM 31 / JCM 2152 / NBRC 15305 / NCIMB 9373 / NRRL B-3711) OX=226900 GN=BC_3103 PE=4 SV=1
**11. rHbl 300 kDa complex**								
Family	Member	Accession	Score	Mass	Num. of Significant Matches	Num. of Significant Sequences	emPAI	Description
2	1	Hbl_B	1220	37838	116	34	83.93	Hbl_B
1	1	Hbl_L2	1392	46140	84	38	59.58	Hbl_L2
3	1	Hbl_L1	1062	40669	71	30	40.57	Hbl_L1
3	2	tr|Q7BYC6|Q7BYC6_BACCR	874	43804	57	27	15.19	Hemolysin BL lytic component L1 OS=*Bacillus cereus* (strain ATCC 14579 / DSM 31 / JCM 2152 / NBRC 15305 / NCIMB 9373 / NRRL B-3711) OX=226900 GN=BC_3103 PE=4 SV=1
**12. rHbl 200 kDa complex**								
Family	Member	Accession	Score	Mass	Num. of Significant Matches	Num. of Significant Sequences	emPAI	Description
1	1	Hbl_L2	3537	46140	167	61	351.31	Hbl_L2
2	1	Hbl_B	2989	37838	165	53	381.85	Hbl_B
3	1	Hbl_L1	1823	40669	81	38	91.72	Hbl_L1
3	2	tr|Q7BYC6|Q7BYC6_BACCR	1367	43804	63	34	29.85	Hemolysin BL lytic component L1 OS=*Bacillus cereus* (strain ATCC 14579 / DSM 31 / JCM 2152 / NBRC 15305 / NCIMB 9373 / NRRL B-3711) OX=226900 GN=BC_3103 PE=4 SV=1

Table S2. Overview of LC-MS experiments. The mass spectrometry proteomics data have been deposited to the ProteomeXchange Consortium [66] via the PRIDE [67] partner repository with the dataset identifier PXD021720.

Band No	Approx. Size (kDa)	Raw Data Name	Measurement
B. cereus supernatant			
1	700	KS 669	No 976 07.02.2020
2	600	KS 440-669	No 976 07.02.2020
3	440	KS 440	No 976 07.02.2020
7	120	KS below 140	No 946 03.12.2019
9	70	KS 66	No 946 03.12.2019
rHbl			
10	440	Hbl 440	No 976 07.02.2020
11	300	Hbl 232-440	No 976 07.02.2020
12	200	rHbl 140-232	No 946 03.12.2019
